# Regulation of miR-200c/141 expression by intergenic DNA-looping and transcriptional read-through

**DOI:** 10.1038/ncomms9959

**Published:** 2016-01-04

**Authors:** Luciana Batista, Brigitte Bourachot, Bogdan Mateescu, Fabien Reyal, Fatima Mechta-Grigoriou

**Affiliations:** 1 Stress and Cancer Laboratory, Equipe Labelisée LNCC, Institut Curie, PSL Research University, 26, rue d'Ulm, Paris 75248, France; 2 Inserm, U830, Paris F-75248, France; 3 Residual Tumor and Response to Treatment Laboratory, Department of Translational Research, Institut Curie, 26, rue d'Ulm, Paris 75248, France

## Abstract

The miR-200 family members have been implicated in stress responses and ovarian tumorigenesis. Here, we find that *miR-200c/141* transcription is intimately linked to the transcription of the proximal upstream gene *PTPN6 (SHP1)* in all physiological conditions tested. *PTPN6* and *miR-200c/141* are transcriptionally co-regulated by two complementary mechanisms. First, a bypass of the regular *PTPN6* polyadenylation signal allows the transcription of the downstream miR-200c/141. Second, the promoters of the *PTPN6* and *miR-200c/141* transcription units physically interact through a 3-dimensional DNA loop and exhibit similar epigenetic regulation. Our findings highlight that transcription of intergenic miRNAs is a novel outcome of transcriptional read-through and reveal a yet unexplored type of DNA loop associating two closely located promoters. These mechanisms have significant relevance in ovarian cancers and stress response, pathophysiological conditions in which miR-200c/141 exert key functions.

## Abstract (web summary)

The miR-141/200 familly of micro RNAs control oxidative stress and impact on ovarian tumorigenesis. Here the authors show that the transcription of miR-200c/141 is regulated through transcriptional read-through of the upstream gene PTPN6, and DNA looping linking the PTPN6 and miR-200c/141 promoters.

## Graphical abstract

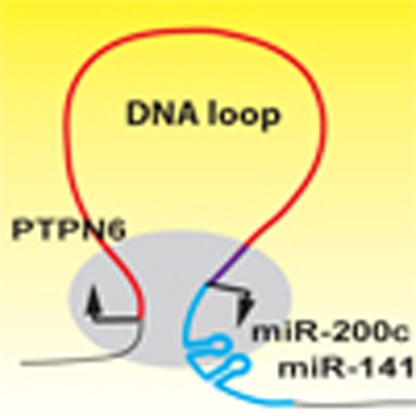

The miR-200 family consists of five microRNAs (miRNAs: miR-141, 200a, 200b, 200c and 429) located in two intergenic genomic clusters on chromosomes 1 and 12 in humans. Several reports highlighted the impact of the miR-200 in cancer development and progression^1^,^2^,^3^,^4^,^5^,^6^,^7^,^8^,^9^,^10^. The miR-200 family regulates key processes in tumorigenesis, such as epithelial–mesenchymal transition^5^,^11^,^12^,^13^,^14^,^15^,^16^,^17^, migration and invasion^18^,^19^,^20^, stem cell maintenance^6^,^21^,^22^,^23^, stromal remodelling^24^,^25^ and oxidative stress response^7^,^26^,^27^,^28^. Moreover, miR-200 expression is induced by oxidative stress in various cells and tissues^7^,^26^,^28^,^29^,^30^,^31^. Finally, these miRNAs accumulate and play a key role in ovarian tumorigenesis and chemosensitivity, the oxidative stress response being one important feature^2^,^4^,^5^,^7^,^9^,^10^,^32^,^33^,^34^,^35^,^36^. Given the important role of miR-200 family members in oxidative stress response and ovarian tumorigenesis, understanding their regulation in these conditions is of major interest. Here we focus on the regulation of the intergenic miR-200 genomic cluster comprising miR-200c and miR-141. Previous studies have identified ZEB1/ZEB2 and p53 transcription factors as regulators of the expression of these miRNAs^11^,^15^,^37^,^38^,^39^. Moreover, epigenetic marks such as DNA and histone methylation have also been linked to miR-200 expression, especially between epithelial and mesenchymal cells^40^,^41^,^42^,^43^. However, despite these efforts, the regulatory mechanisms involved in miR-200 expression are still far from being completely understood.

In contrast to intronic miRNAs that are frequently transcribed along with their host genes, it is widely assumed that the regulation of intergenic miRNAs, such as miR-200c and miR-141, relies on their own promoters^44^,^45^. We show here that transcription of intergenic miRNAs can also be modulated by their surrounding genes. Indeed, alternative polyadenylation (APA) and transcriptional read-through of an upstream gene can be new means of producing intergenic miRNAs. APA concerns about half of the human genes. APA shortens or lengthens the 3′-extremity of messenger RNAs and subsequently affects mRNA stability or protein translation^46^. Although it has not yet been described to date, a new outcome for APA could be the transcription of miRNAs located between two alternative polyA sites. Another yet unexplored mechanism that could also participate in the regulation of intergenic miRNA expression is a chromatin interaction between its promoter and another regulatory locus. Recent reports underline the functionality of both long-range and short-range chromatin contacts^47^,^48^,^49^. Short-range DNA loops can bring together the promoter and terminator regions of the same gene. This type of DNA conformation, called ‘gene-loop', plays a role in transcriptional memory and directionality and has been identified from yeast to mammals^50^. However, the role of such DNA interactions on intergenic miRNA regulation has not been established so far.

Here, we show evidence that the miR-200c/141 intergenic cluster is regulated through two original complementary mechanisms: the read-through of the transcription of a neighbouring gene and the three-dimensional (3D) chromatin interaction involving the miRNA promoter region. We demonstrate that the transcription of miR-200c/141 is tightly associated with that of *PTPN6* (also called *SHP1*), a gene whose 3′ extremity is located less than 3 kilobases upstream of the miRNAs. We show that the bypass of the usual *PTPN6* polyadenylation signal can lead to the transcription of miR-200c/141, and we reveal the existence of a 3D DNA conformation associating the promoters of these two genes. Together, our data indicate a physiological relevance of *PTPN6* expression in *miR-200c/141* regulation in all tested situations. We propose that the systematic correlation between these two genes in several different contexts is due to transcriptional read-through of the *PTPN6* gene and a previously unexplored type of DNA loop associating two closely located promoters.

## Results

### miR-200c and miR-141 are regulated at transcriptional level

**Figure 1:**
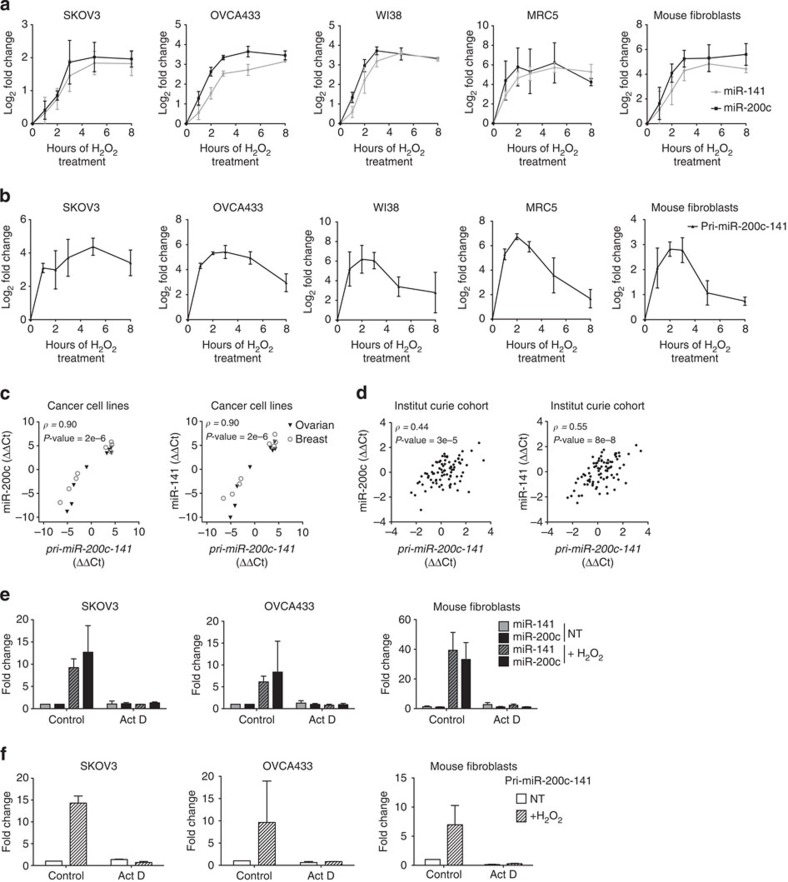
Transcriptional regulation is crucial for miR-200c and miR-141 expression. (**a**,**b**) Kinetics of accumulation of miR-200c (black) and miR-141 (grey; **a**) and their corresponding primary transcript, *pri-miR-200c-141* (**b**), following H_2_O_2_ treatment in human ovarian cancer cell lines (SKOV3, OVCA433), human (WI38, MRC5) and mouse fibroblasts, as indicated. qRT–PCR data are means of fold changes (normalized to untreated and expressed as log_2_)±s.d. *n*=3 independent experiments at least, except for OVCA433, *n*=2 (in this case error bars indicate range of data). Kinetics of accumulation in eight other cell lines can be found in Supplementary Fig. 1. Sequences of the pri-miR-200c-141F/R primers from mouse or human origins are given in Supplementary Table 2. (**c**,**d**) Scatter plots showing that levels of miR-200c (left) and miR-141 (right) are correlated with *pri-miR-200c-141* primary transcript levels in ovarian (IGROV-1, OV90, OV2008, OVCAR3, OVCA433, RMG1, SHIN3, SKOV3) and breast (SKBr3, MDA-MB-468, MCF7, MDA-MB-231, MDA-MB-436, MDA-MB-453, BT549) cancer cell lines (**c**) and in HGSOC (**d**). qRT–PCR data are shown as normalized cycle threshold centred to the mean (ΔΔCt). Spearman correlation coefficients *ρ* (rho) and corresponding *P-*values are indicated on each graph. *n*=3 independent experiments per cell line, except for OVCAR3 cells, *n*=2. (**e**,**f**) Effect of the transcription inhibitor actinomycin D (Act D) on the levels of miR-141 (grey) or miR-200c (black) (**e**) and their corresponding primary transcript, *pri-miR-200c-141* (**f**), under untreated conditions (NT) or following 3 h of H_2_O_2_ treatment (+H_2_O_2_). Cells were pretreated for 4 h with Act D or dimethylsulphoxide (control). qRT–PCR data are shown as fold change±s.d. of treated compared with untreated cells. *n≥*2 independent experiments per cell line.

Regulation of miR-200c and miR-141 (hereafter referred to as miR-200c/141) expression represents a crucial step in ovarian tumorigenesis and stress response. A better understanding of their regulation deserved further investigation in these two related pathophysiological conditions. As both miR-200c and miR-141 are transcribed from the same genomic locus, we first observed that the levels of the two mature miRNAs were directly proportional to their corresponding primary transcript (below referred to as *pri-miR-200c-141*) in the diverse conditions analysed, that is, following acute oxidative stress and in high-grade ovarian carcinomas (HGSOC; Fig. 1a–d and Supplementary Fig. 1a,b). Indeed, the kinetics of miR-200c/141 accumulation after exposure to H_2_O_2_ followed the increase of *pri-miR-200c-141* transcript in epithelial or fibroblast cell lines from both human or mouse origin (Fig. 1a,b and Supplementary Fig. 1a,b). We detected oxidative stress-mediated miR-200c/141 upregulation in all cell lines tested, except those already expressing high basal concentrations of the miR-200c/141, such as IGROV-1 cell line (Supplementary Fig. 1d). Similarly, basal levels of mature and primary miR-200c/141 were found significantly correlated in different ovarian and breast cancer cell lines (Fig. 1c) as well as in a cohort of 107 human primary HGSOC (Fig. 1d and Supplementary Table 1 for description of the cohort). This suggested that the accumulation of miR-200c and miR-141 relies mainly on the regulation of their transcription, rather than on maturation steps. Accordingly, treatment with actinomycin D, an effective transcriptional inhibitor, abolished the increase of miR-200c and miR-141 upon H_2_O_2_ exposure (Fig. 1e,f). Thus, our data suggest that transcription is a major regulatory step controlling the accumulation of mature miR-200c/141.

### miR-200c/141 transcription is coupled to *PTPN6* expression

**Figure 2:**
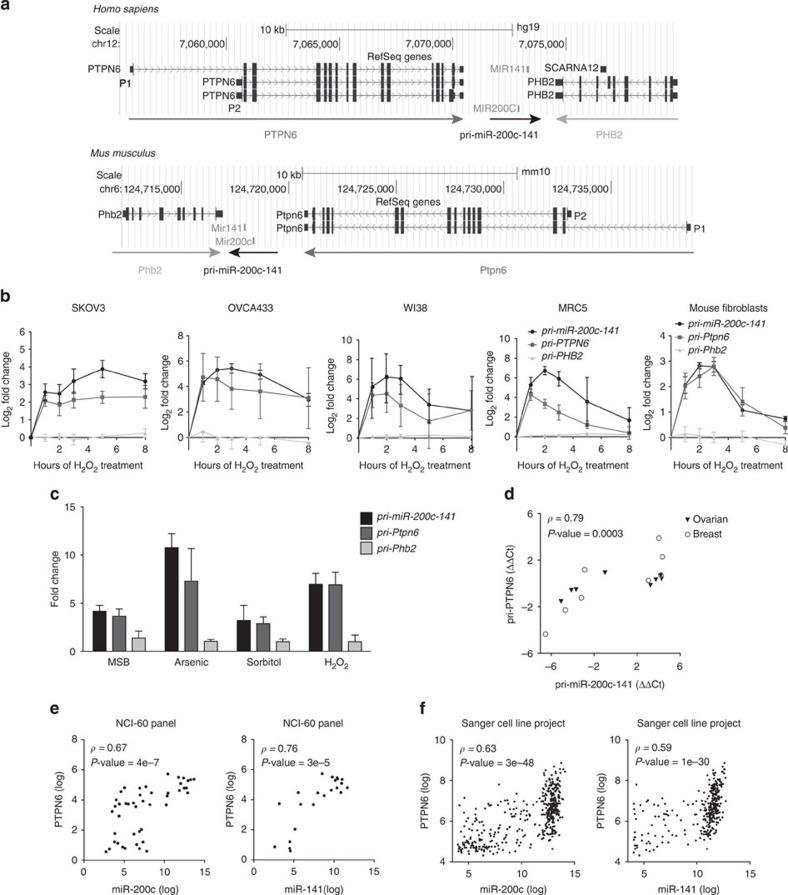
*pri-miR-200c-141* expression correlates with *PTPN6* transcription. (**a**) Schematic representation (adapted from http://genome.ucsc.edu) of miR-200c/141 genomic locus showing upstream (*PTPN6*) and downstream (*PHB2, SCARNA12*) neighbouring genes in human and mouse species, as indicated. Genomic positions, scale and genome versions used (*hg19* and *mm10*) are indicated on the top. Horizontal arrows indicate the sense of transcription. Coding exons are indicated as blocks connected by horizontal lines representing introns, and arrowheads showing sense of transcription. Untranslated regions are presented by thinner blocks. P1 and P2 indicate the alternative promoters used for *PTPN6* transcription in non-haematopoietic and haematopoietic cells, respectively. (**b**) Kinetics of accumulation of *pri-miR-200c-141, pri-PTPN6* and *pri-PHB2* primary transcripts following H_2_O_2_ treatment in human ovarian cancer cells (SKOV3, OVCA433), human (WI38, MRC5) and mouse fibroblasts. qRT–PCR data are means of fold changes (normalized to untreated and expressed as log_2_)±s.e.m. *n*=3 independent experiments at least, except for OVCA433, *n*=2 (in this case error bars indicate range of data). Sequences of the primers from mouse or human origins (pri-miR-200c-141F/R and pri-PTPN6/Ptpn6(2) F/R) used are given in Supplementary Table 2. (**c**) Effect of different stresses on *pri-miR-200c-141, pri-Ptpn6* and *pri-Phb2* primary transcript levels. MSB, menadione sodium bisulfite. qRT–PCR data are shown as fold change±s.d. following treatments, compared with untreated fibroblasts (NT). *n*=3 independent experiments. (**d**) Scatter plot showing correlation between *pri-PTPN6* and *pri-miR-200c-141* primary transcript levels in ovarian and breast cancer cells. qRT–PCR data are shown as normalized cycle threshold centred to the mean (ΔΔCt). (**e**,**f**) Scatter plots showing that miR-200c (left) and miR-141 (right) levels are correlated with *PTPN6* mRNA levels in NCI-60 panel of cell lines (**e**) and Sanger Cell Line Project (**f**). Spearman correlation coefficients *ρ* (rho) and *P-*values are indicated on each graph.

We first observed that neither ZEB nor TP53 protein was involved in the upregulation of miR-200c/141 by oxidative stress in ovarian cancer cell lines (Supplementary Fig. 1c), suggesting that the mechanism involved was complementary to those previously described. We thus analysed the genomic locus from which these miRNAs are transcribed (Fig. 2a). We first tested whether transcription of the miR-200c/141 neighbouring genes could be affected by acute oxidative stress. We observed that the transcription of the immediate upstream gene, *PTPN6*, was significantly increased following H_2_O_2_ treatment, whereas the downstream gene, *PHB2*, remained invariant (Fig. 2b). Interestingly, the kinetics of *PTPN6* upregulation was strikingly similar to this one of the miR-200c/141 primary transcript, *pri-miR-200c-141* (Fig. 2b). The coupling between *PTPN6* and *miR-200c/141* primary transcripts upon stress was observed in 12 different cell lines (Fig. 2b and Supplementary Fig. 1b), suggesting a conserved mechanism of co-regulation in mammals. Moreover, this coupled transcription was also observed upon treatments increasing reactive oxygen species (Fig. 2c). Furthermore, the remarkable association between *pri-miR-200c-141* and *pri-PTPN6* transcription was not restricted to stress conditions but also observed at basal state in ovarian and breast cancer cell lines (Fig. 2d). We further confirmed this result by validating the correlation between *PTPN6* and miR-200c/141 levels in publicly available data sets from two large cell line panels, the NCI60 panel and the Sanger Cell Line Project (Fig. 2e,f). Taken together, these results demonstrate that miR-200c/141 and *PTPN6* transcriptions are tightly associated in a variety of cell lines at basal state and upon oxidative stress.

### *Pri-miR-200/PTPN6* transcripts are coupled in ovarian cancer

**Figure 3:**
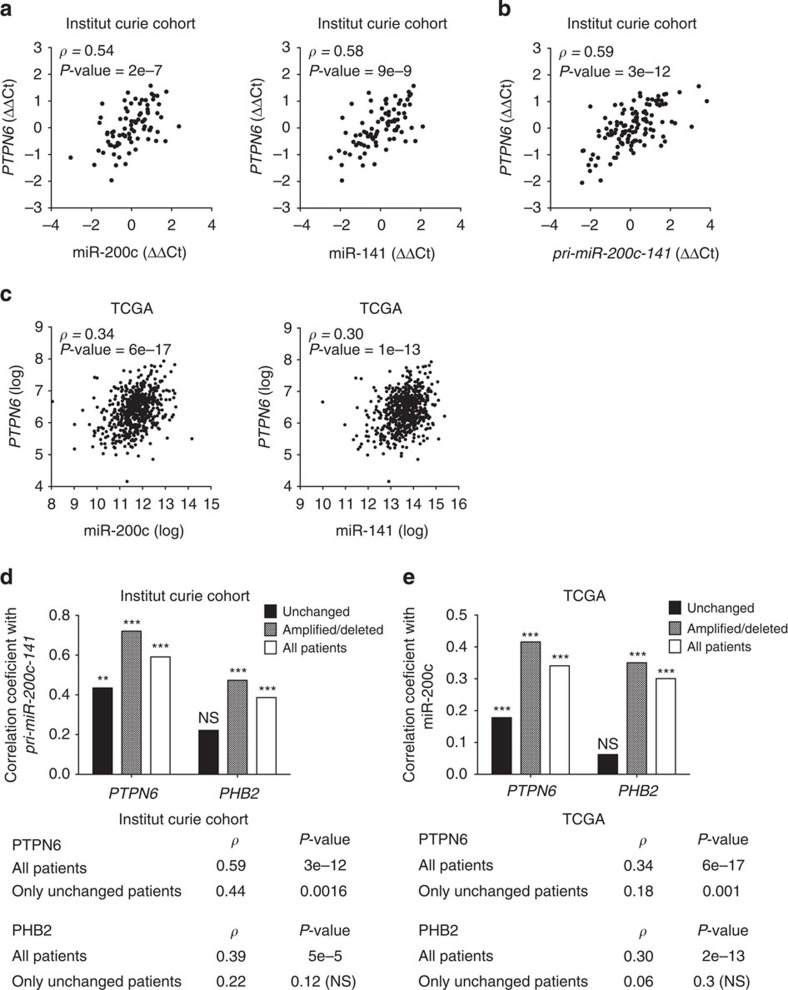
*miR-200c/141* expression correlates with *PTPN6* in HGSOC, independently of genomic alterations. (**a**) Scatter plots showing that *PTPN6* mRNA levels are correlated with miR-200c (left) and miR-141 (right) levels in ovarian tumours (Institut Curie cohort). qRT–PCR data are shown as normalized cycle threshold centred to the mean (ΔΔCt). (**b**) Scatter plot showing correlation between levels of *PTPN6* and *pri-miR-200c-141* primary transcripts in ovarian tumours (Institut Curie cohort). qRT–PCR data are shown as normalized cycle threshold centred to the mean (ΔΔCt). Sequences of the primers (*pri-miR-200c-141F/R* and *pri-PTPN6(1) F/R*) used for detection of primary transcripts are given in Supplementary Table 2. (**c**) Scatter plots showing that *PTPN6* mRNA levels are correlated with miR-200c (left) and miR-141 (right) levels in ovarian tumours of the TCGA cohort. Microarray data were obtained from the TCGA portal. *pri-miR-200c-141* levels were not available. (**d**,**e**) Correlation coefficients between *PTPN6* or *PHB2* mRNA levels with *pri-miR-200c-141* primary transcript levels in ovarian tumours from the Institut Curie (**d**) and TCGA (**e**) cohorts. Correlations take into account either all patients (white) or patient subgroups defined according to their genomic status: amplified/deleted (grey), patients with amplifications or deletions in the region of interest; unchanged (black), patients with no genomic alteration in the region of interest. Numbers below indicate Spearman correlation coefficients *ρ* (rho) and corresponding *P-*values per gene (*PTPN6* or *PHB2*) for each subgroup of patients in each cohort. NS, not significant.

As miR-200c/141 have been repeatedly defined as key regulators of human ovarian tumorigenesis^5^,^7^,^9^,^10^,^15^,^32^,^33^,^34^,^35^,^36^, we next wondered whether the correlation of expression between miR-200c/141 and *PTPN6* was also observed in HGSOC. We first observed that the expression of the two miRNAs (Fig. 3a) and their corresponding primary transcript (Fig. 3b) were correlated with *PTPN6* mRNA levels in a cohort of 107 HGSOC (Fig. 3a,b). Correlation between miR-200c/141 and *PTPN6* expression was further validated in the publicly available The Cancer Genome Atlas (TCGA) database^51^ composed by 574 HGSOC patients (Fig. 3c and details of the cohort are given in Supplementary Table 1).

Genomic amplifications have been previously described in HGSOC on the chromosome 12p13, including the genomic locus from which the *miR-200c/141* and *PTPN6* are transcribed^51^. We hypothesized that genomic amplification could contribute to the observed correlation between *PTPN6* and *pri-miR-200c-141* transcription levels in these tumours. We thus computed the Transcription Correlation Scores (TCS) of this genomic region (Supplementary Fig. 2), TCS reflecting the degree of correlation of each gene with its neighbours, as defined previously^52^. We observed a peak of correlation in the TCS map around the *PTPN6* gene in both Institut Curie and TCGA cohorts (Supplementary Fig. 2a,b). We analysed the copy number alterations of HGSOC samples and classified the patients, referred to as unchanged or amplified/deleted, according to their genomic status in this specific region (Supplementary Fig. 2e). When we restricted the analysis to patients of the unchanged subgroup, the TCS peak observed around the *PTPN6* gene with all patients was lost in both cohorts (Supplementary Fig. 2c,d), revealing this peak was indeed because of genomic alterations. In contrast, the correlation between *PTPN6* and *pri-miR-200c-141* transcription was maintained in patients with no copy number alteration in this locus (unchanged subgroup; Fig. 3d). Interestingly, in this subgroup, the correlation was restricted to *PTPN6*, and was not observed with *PHB2*, the downstream gene (Fig. 3d). Accordingly, the same findings were obtained from the TCGA cohort (Fig. 3e), strengthening the validity of such data. In this cohort, mature miRNA levels were analysed, as *pri-miRNA-200c-141* levels were not available. It is noteworthy that *PTPN6* was ranked the 13th-most correlated gene with miR-200c among the 12,044 genes tested, in the TCGA cohort. Together, these results indicate that the transcriptional co-regulation between *PTPN6* and miR-200c/141 is a relevant process for the expression of the two miRNAs in HGSOC independently of genomic alterations.

### Two complementary mechanisms drive miR-200c/141 expression

To explain the tight correlation between *pri-miR-200c-141* and *PTPN6* primary transcripts, we considered two non-exclusive hypotheses (Supplementary Fig. 2f). In the first hypothesis, the *PTPN6* primary transcription bypasses its usual polyadenylation site, thereby promoting the downstream expression of miR-200c/141. In this case, *miR-200c/141* transcription results from either an APA of *PTPN6* mRNA or a late termination of *PTPN6* transcription. In the second hypothesis, *pri-PTPN6* and *pri-miR-200c-141* primary transcripts are produced from two independent but co-regulated promoters. This co-regulation could be mediated by a spatial DNA conformation, favouring shared regulation by common transcription factors and/or epigenetic marks. The two mechanisms could work together, with PTPN6 bypass favouring the transcription initiation at the miRNA locus, for example. It is worth noting that the *PTPN6* gene has two alternative promoters (Fig. 2a). Here, we were interested in the most 5′ one, also called P1, which is the one used in almost all cell types, except haematopoietic cells, in which P2 is active 53. We will refer below to *PTPN6* P1 promoter as *PTPN6* promoter.

### The polyadenylation site of the *PTPN6* gene is bypassed

**Figure 4:**
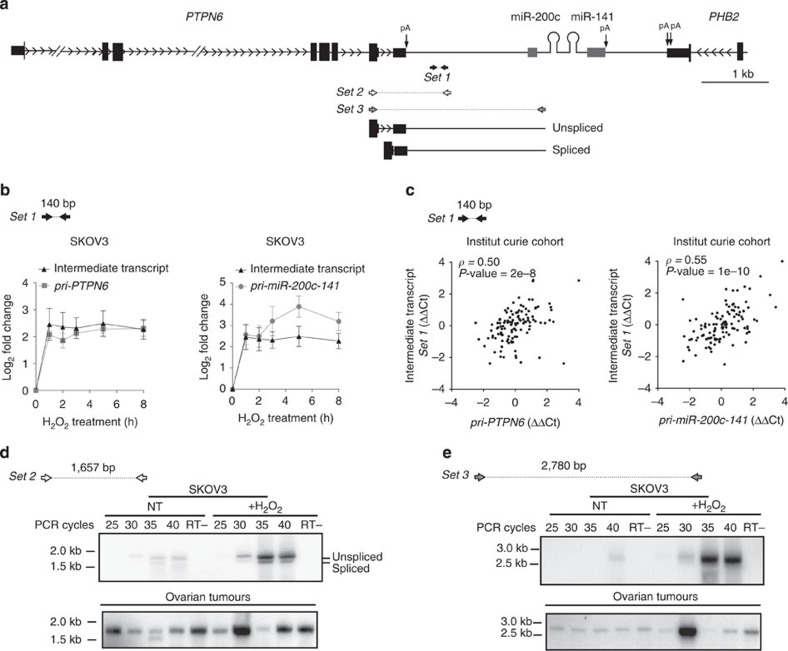
The usual polyadenylation site of the *PTPN6* gene is bypassed. (**a**) Schematic representation (adapted from UCSC browser: https://genome.ucsc.edu) of the human genomic locus showing *miR-200c/141* and their neighbouring genes, *PTPN6* and *PHB2*. Black and grey boxes represent exons and ESTs, respectively. Polyadenylation sites (pA) are indicated (see details in Supplementary Fig. 5). *Set 1*, *Set 2* and *Set 3* primers allow detection of the Intermediate transcript by qPCR (*Set 1*) and by PCR (*Set 2*, *Set 3)*, respectively. Sequences are given in Supplementary Table 2. Lengths of the amplified products using *Set 1–3* primers are indicated in base pairs (bp) in **b**,**d**,**e**. (**b**) Kinetics of accumulation of the intermediate transcript detected using *Set 1* primers upon H_2_O_2_ treatment in SKOV3 ovarian cancer cells and compared with *pri-PTPN6* (left) or *pri-miR-200c/141* (right) primary transcripts. qRT–PCR data are means of fold changes (normalized to untreated and expressed as log_2_)±s.e.m. *n*≥3 independent experiments. (**c**) Correlation plot showing that RNA levels of the intermediate transcript are correlated with *pri-PTPN6* (left) and *pri-miR-200c-141* (right) primary transcript levels in ovarian tumours (Institut Curie cohort). (**d**,**e**) PCR reactions using *Set 2* (**d**) and *Set 3* (**e**) primers showing intermediate transcripts spanning from *PTPN6* 3′-end and reaching miR-200c/141 locus. Representative amplifications using cDNA from untreated (NT) or H_2_O_2_-treated (+H_2_O_2_) SKOV3 cells (up) and ovarian tumours (bottom). Numbers of PCR cycles performed are indicated on the top. RT- indicates control without the reverse transcriptase enzyme. The amplified fragments have been cloned and sequenced to verify that they correspond to the intermediate transcript.

We first demonstrated the existence of a transcript, downstream of the polyadenylation site of the *PTPN6* gene and upstream of the miR-200c/141-coding region (schema Fig. 4a). This intermediate transcript (assessed with *Set 1* primers, compatible with quantitative reverse transcription–PCR (qRT–PCR) experiments) accumulated with the same kinetics as *PTPN6* primary transcript upon oxidative stress (Fig. 4b, left panel, and Supplementary Fig. 3a,b, left panel). As expected according to the clear correlation between *PTPN6* and *pri-miR-200c-141*, this intermediate transcript followed also the same kinetics as *pri-miR-200c-141* (Fig. 4b, right panel, and Supplementary Fig. 3b, right panel). We thus speculated that this intermediate transcript was the result of a transcriptional read-through from the *PTPN6* gene. In agreement with the assumption that *PTPN6* read-through led to the transcription of the downstream miR-200c/141, this intermediate transcript was also correlated with the *pri-miR-200c-141* in HGSOC (Fig. 4c, right). Moreover, we also detected a significant correlation between the expression levels of this intermediate transcript and *PTPN6* primary transcript in HGSOC (Fig. 4c, left). Importantly, a read-through from the *PTPN6* gene was also detected in RNA-seq data generated from a set of cell lines in the ENCODE project and visualized using the UCSC Genome Browser (Supplementary Fig. 4). The levels of expression of the intermediate transcript, while being much lower than mature (exonic) *PTPN6* mRNA as expected, reached the same expression rate as the *pri-PTPN6* primary (intronic) transcript (Supplementary Fig. 4). We next demonstrated that this intermediate transcript resulted from the bypass of the usual polyadenylation site of the *PTPN6* gene (Fig. 4d,e). Indeed, we detected both spliced and unspliced transcripts extending from *PTPN6*-coding sequence to the downstream region of its usual polyadenylation site, in both mouse and human cells at basal states and following H_2_O_2_ exposure (*Set 2* primers, Fig. 4d and Supplementary Fig. 3c). Importantly, the same type of transcript was also detected in human HGSOC (Fig. 4d, bottom). Furthermore, we confirmed that these bypassed *PTPN6* transcripts reached the *pri-miR-200c-141* sequence both in cell lines and in HGSOC (*Set 3* primers, Fig. 4e and Supplementary Fig. 3d), thus showing that *PTPN6* read-through could indeed reach the miR-200c/141 locus and impact directly *pri-miR-200c-141* expression. The products amplified using the *Set 2* and *Set 3* couples of primers were cloned and sequenced, in order to verify that they corresponded to the region located between *PTPN6* 3′-end and *miR-200c-141* 5′-end. It was noteworthy that this intermediate transcript was detected at the same levels by quantitative PCR (qPCR) by using either random hexamers or oligo dT in the reverse transcription reaction, suggesting it was polyadenylated. Consistently, using the 3′ Rapid Amplification of cDNA Ends (RACE) methodology, we identified APA sites reached by the *pri-PTPN6/pri-miR-200c-141* transcript (Supplementary Fig. 5a,b). These observations thus demonstrate that the read-through transcription from the *PTPN6* gene can reach the *miR-200c-141* encoding unit and that the levels of these transcripts are comparable. According to these results, the transcriptional co-regulation between *PTPN6* and *miR-200c-141* genes can result from late termination of transcription by RNA-polymerase II or APA (hypothesis 1, Supplementary Fig. 2f).

### *PTPN6* read-through drives *pri-miR-200c-141* transcription

**Figure 5:**
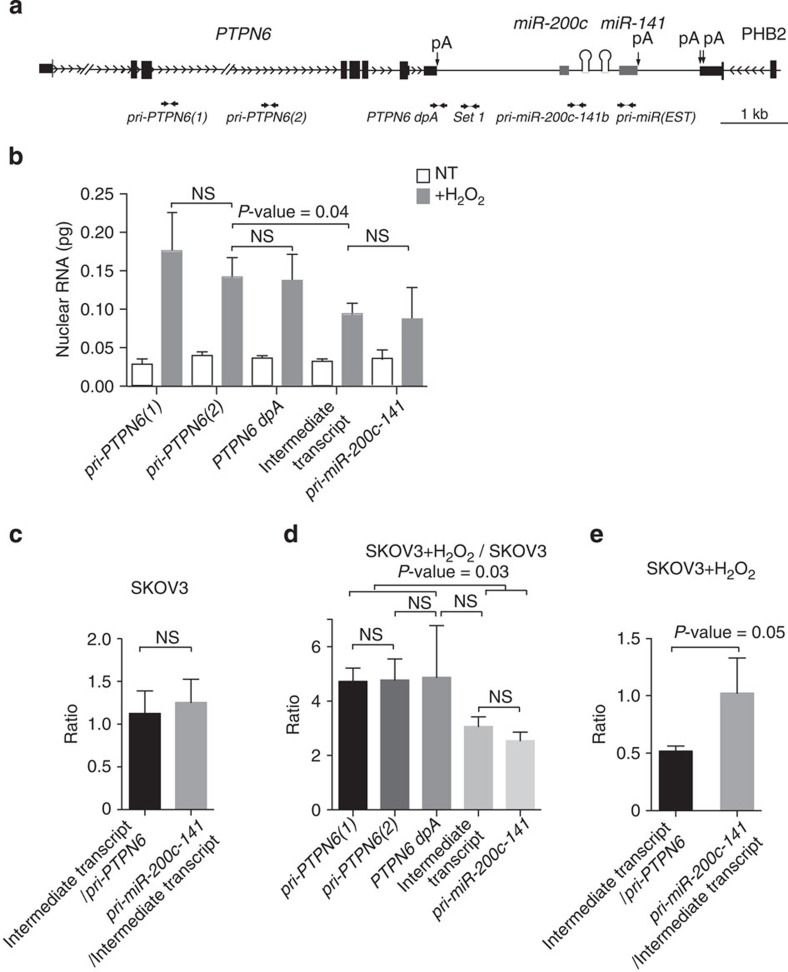
Quantification of primary transcripts detected along *PTPN6* and *miR-200c/141* genomic locus. (**a**) Schematic representation of the human *PTPN6-miR-200c/141* genomic locus, as shown and explained in Fig. 4a. (**b**) Quantities of primary transcripts detected along *PTPN6* and *miR-200c-141* genomic locus, assessed by qPCR using cDNA from nuclear RNA of untreated (NT) or H_2_O_2_-treated SKOV3 cells. *pri-PTPN6(1)* and *pri-PTPN6(2)* primers detect the *pri-PTPN6* primary transcript, *PTPN6 dpA* overlaps *PTPN6* polyadenylation signal, *Set 1* allows detection of the Intermediate transcript and *pri-miR-200c-141b* detects the *pri-miR-200c-141* primary transcript. Quantifications were assessed using standard curves shown in Supplementary Fig. 6. Quantities are expressed in picogram (pg). Data are means of±s.e.m. *n*=6 independent experiments per cell line. *P*-values are from Student's *t*-test. (**c**,**e**) Ratios of quantities of intermediate transcript versus *pri-PTPN6* primary transcript and *pri-miR-200c-141*/Intermediate transcript, as indicated in SKOV3 cells (**c**) and SKOV3+H_2_O_2_ (**e**). (**d**) Ratios of quantities of each primary transcript (as indicated) in SKOV3+H_2_O_2_ versus SKOV3 cells. *P*-values are from Student's *t*-test. Data are means *n*=6 independent experiments. NS, not significant.

We next wondered in which proportion the read-through from the *PTPN6* gene participates to *miR-200c/141* transcription. We quantified the expression rates of each RNA entity detected along *PTPN6* and *miR-200c-141* genomic locus, namely the *pri-PTPN6* primary transcript, the intermediate transcript (RNA detected downstream *PTPN6* polyadenylation signal and upstream *miR-200c/141* promoter) and the *pri-miR-200c-141* primary transcript, whose synthesis could result from both *PTPN6* read-through and transcription from miR-specific promoter (Fig. 5a). Two experimental proceedings were adopted to quantify these weakly expressed molecules. First, nuclear RNA was used, in order to increase sensitivity of the assay. Second, *pri-miR-200c-141* amount was evaluated using qPCR primers located outside of a previously described expressed sequence tag (EST) (grey boxes Fig. 5a). This avoided bias related to different RNA stability (when comparing EST and intronic RNA) and allowed comparison with intronic *pri-PTPN6* and intermediate transcript in absolute quantities. Based on standard curves obtained on serial dilutions of a bacterial artificial chromosome (BAC) DNA corresponding to the same genomic locus (Supplementary Fig. 6a), we first observed that the quantity of the intermediate transcript was equivalent to the level of the *pri-PTPN6* and *pri-miR-200c-141* primary transcripts in SKOV3 ovarian cancer cells at basal state (Fig. 5b). Indeed, in these cells, although the expression rate was faint, data indicated that all molecules of *pri-PTPN6* primary transcript were able to bypass *PTPN6* polyadenylation signal (ratio *Intermediate transcript/pri-PTPN6*=1; Fig. 5c). Moreover, all intermediate transcripts gave rise to *pri-miR-200c-141* primary transcripts (ratio *pri-miR-200c-141/Intermediate transcript*=1; Fig. 5c; see also Fig. 9a for schematic representation of the data). Upon H_2_O_2_ exposure, *PTPN6* transcription initiation (evaluated by the *pri-PTPN6* transcript quantity) increased fivefold (Fig. 5d), whereas the levels of the intermediate transcript was only induced threefold (Figs 5d,e). Indeed, only 56% of *PTPN6* primary transcripts were able to bypass *PTPN6* polyadenylation signal, suggesting that *PTPN6* read-through was reduced by oxidative stress. Still, the totality of the 56% intermediate transcript contributed to *pri-miR-200c-141* primary transcription (ratio *pri-miR-200c-141/Intermediate transcript*=1) in SKOV3 following oxidative stress (Fig. 5e).

**Figure 6:**
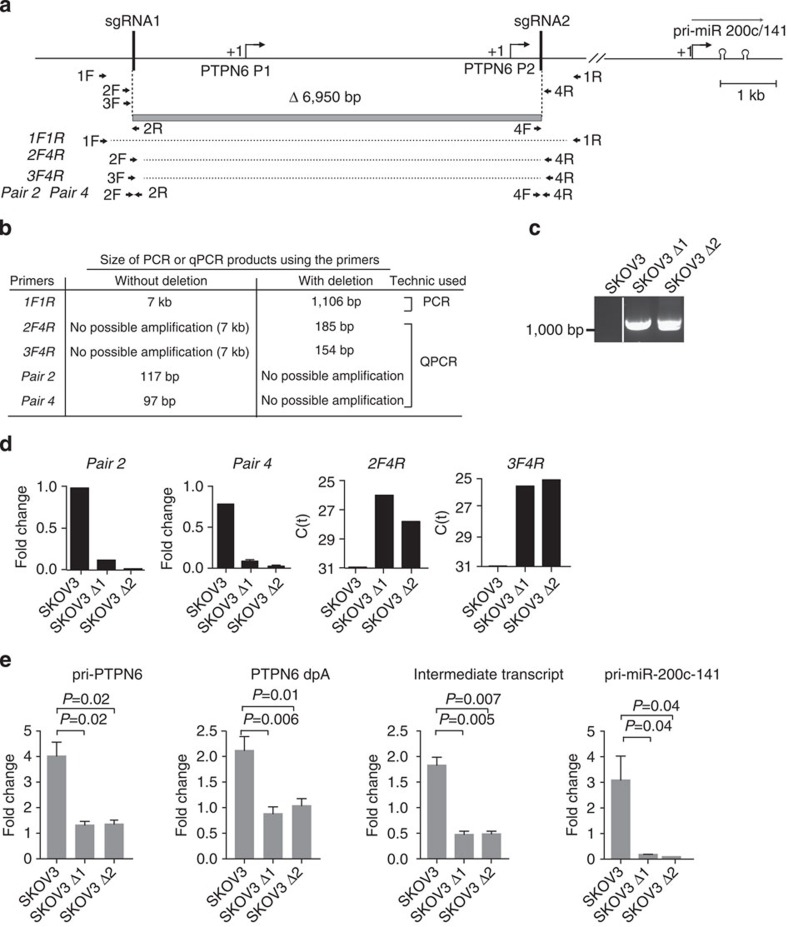
*PTPN6* transcription is necessary for *pri-miR-200c-141* transcription. (**a**) Schematic representation of the genomic deletion of 6,950 bp (grey box) targeting the *PTPN6* promoter region using the CRISPR/Cas9 technology. Are represented the P1 and P2 promoters, expressed in epithelial and haematopoietic cells, respectively, as well as the position of the target-specific CRISPR guide RNAs (sgRNA). Double arrows represent the position and orientation of the primers used. (**b**) Table summarizing the sizes of the PCR- and qPCR-amplification products used for genotyping. (**c**,**d**) Characterization of 2 SKOV3-derived cell lines (SKOV3-Δ1, SKOV3-Δ2) among 63 individual clones tested after depletion of *PTPN6* promoter genomic region using the CRISPR/Cas9 technology. (**c**) Fragment of 1,106 pb amplified by PCR from genomic DNA of SKOV3-Δ1 and SKOV3-Δ2 cell lines using *1F1R* primers. As expected, no amplification was seen in parental SKOV3 cells. (**d**) qPCR-based genotyping results from genomic DNA of SKOV3, SKOV3-Δ1 and SKOV3-Δ2 cell lines. Amplified products obtained from primers described in **b**. *Pair 2* and *Pair 4* primers are specific of the deleted genomic region; fragments from 2F4R and 3F4R primers can be amplified only in case of deletion. qPCR data are shown as fold change normalized to SKOV3 for *Pair2* and *Pair4* primers and as normalized cycle threshold for *2F4R* and *3F4R* primers, as the signal is null in SKOV3 parental cells. (**e**) qRT–PCR data showing *pri-PTPN6*, *PTPN6 dpA*, *intermediate transcript* and *pri-miR-200c-141* primary transcripts. Data are from SKOV3, SKOV3-Δ1 and SKOV3-Δ2 cell lines, following 3 h of H_2_O_2_ treatment. Data are means of fold changes (normalized to untreated and expressed as log_2_)±s.e.m. *n*=3 independent experiments per cell line. *P*-values are from Student's *t*-test.

Finally, we evaluated the role of *PTPN6* transcription and read-through in stress-induced *pri-miR-200c-141* transcription by using the CRISPR/Cas9 technology (Fig. 6). In that aim, we deleted the genomic region containing the two promoters (P1 and P2) driving *PTPN6* transcription in epithelial and haematopoietic cells, respectively (Fig. 6a). We decided to avoid the use of a selection gene, which would have required the insertion of an independent promoter with the risk of an ectopic transcriptional start in the *PTPN6* locus. We selected two stable cell lines from parental SKOV3 cells (SKOV3-Δ1 and SKOV3-Δ2) based on PCR and qPCR genotyping (Fig. 6b–d). Genomic deletion of *PTPN6* promoters reduced significantly *PTPN6* upregulation by oxidative stress (Fig. 6e, left), as expected for a transcriptional-dependent regulation. Importantly, depletion of *PTPN6* promoters also prevented the induction of both the intermediate transcript and the *pri-miR-200c-141* transcript (Fig. 6e, middle and right). These experiments demonstrate that *PTPN6* transcription is required for *pri-miR-200c-141* upregulation upon stress. Taken as a whole, these results show that the transcriptional read-through from the *PTPN6* gene leads to the transcription of the *pri-miR-200c-141* and explains, at least in part, how *PTPN6* transcription could regulate miR-200c/141 levels following oxidative stress in ovarian cancer cells.

### *PTPN6* and *miR-200c/141* promoters share epigenetic marks

**Figure 7:**
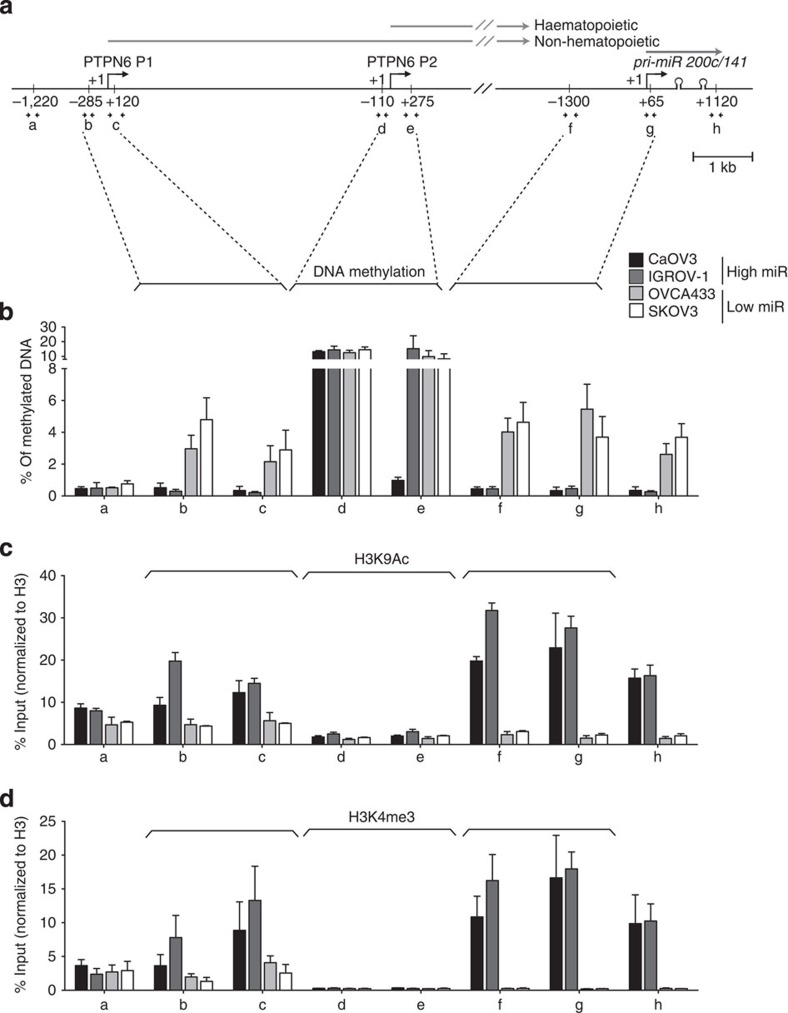
The same epigenetic marks are associated with *PTPN6* and *miR-200c/141* promoters. (**a**) Schematic representation of *PTPN6* and *pri-miR-200c-141* genomic organization showing the localization of the primers (referred to as a–h) used for MeDIP and ChIP experiments. Broken arrows represent *miR-200c/141* and *PTPN6* promoters, PTPN6-P1 and PTPN6-P2 being active in non-haematopoietic and haematopoietic cells, respectively. +1 indicate the start sites of *PTPN6* and *miR-200c/141* transcription. Positions of the primers relative to each start site are indicated. Sequences of the primers are listed in Supplementary Table 2. (**b**) Percentage of methylated DNA, defined by MeDIP experiments using primers indicated in **a** in ovarian cancer cell lines characterized by high (CaOV3, IGROV-1) or low (OVCA433, SKOV3) expression of *PTPN6* and *miR-200c/141*, as indicated. Values are means±s.e.m. *n*=6 independent experiments per cell line, except for CaOV3, *n*=2 (in this case error bars indicate range of data). (**c**,**d**) ChIP experiment using anti-H3K9Ac- (**c**) and H3K4me3-specific antibodies (**d**) in ovarian cancer cell lines, as indicated. Values are presented as percentage (%) of input DNA (amount of DNA extracted per experiment), normalized to DNA amount immunoprecipitated using anti-histone H3 antibody. Values are means±s.e.m. *n*=3 independent experiments at least per cell line, except for CaOV3, *n*=2 (in this case error bars indicate range of data).

As described above, we observed a strict correlation between *PTPN6* and *pri-miRNA-200c-141* transcription. It has been previously shown that *miR-200c/141* transcription can be initiated downstream from the *PTPN6* gene and regulated by an independent promoter^11^,^15^,^37^,^38^,^39^. In addition to the read-through of the *PTPN6* gene, we thus next checked whether the transcription of *pri-miR-200c-141* could be initiated at its closest transcriptional start site (TSS). Indeed, *PTPN6* bypass could reach miR-200c/141 locus but also favour transcription initiation of poised RNA Polymerase II on miRNA TSS. Using 5′RACE method, we were able to confirm the existence of a capped mRNA entity, whose 5′-extremity was compatible with previously identified EST located upstream of miR-200c (Supplementary Fig. 5a). Consistently, chromatin immunoprecipitation (ChIP) experiments confirmed RNA polymerase II recruitment at *PTPN6* P1 and *miR 200c/141* promoters (Supplementary Fig. 5c,d). Thus, in addition to transcripts generated through the bypass of the *PTPN6* polyA site, *miR-200c/141* expression can also be initiated at their independent promoter. As we observed a striking correlation between *PTPN6* and *pri-miR-200c-141* transcription, we next wondered if the two promoters could share regulatory mechanisms. By performing methylated-DNA immunoprecipitation (MeDIP) experiments, we showed that *PTPN6*- and *miR-200c/141*-specific promoters were both methylated in cell lines with low expression of miR-200c/141 and demethylated in cells characterized by high-miR-200c/141 expression (Fig. 7a,b), confirming previous results^40^,^42^,^43^. Accordingly, both *PTPN6* and *pri-miRNA-200c-141* genes were upregulated upon treatment with a DNA demethylating agent, 5-aza-2'-deoxycytidine (Supplementary Fig. 7a). As expected, the *PTPN6* haematopoietic promoter P2 is highly methylated in ovarian cancer cells. Moreover, the active histone methylation marks H3K9Ac and H3K4me3 enrichment were in complete agreement with DNA methylation status in all cell lines tested (Fig. 7c,d). Together, these results indicate that the *PTPN6* and *miR-200c/141* promoters could be under equivalent epigenetic regulation. We further extended this observation to 46 non-haematopoietic cell lines of the ENCODE project, in which the methylation states of *PTPN6* and *miR-200c/141* promoters were correlated (Supplementary Fig. 7b). Finally, in HGSOC from the TCGA cohort, although miR-200c/141 expression levels were globally high, the methylation status of the *PTPN6* promoter was significantly negatively correlated with the miR-200c/141 expression levels (Supplementary Fig. 7c), highlighting the relevance of our findings not only in cultured cell lines but also in human cancers. In conclusion, *PTPN6* and *miR-200c/141* regulatory regions exhibit similar epigenetic marks and might share epigenetic-regulatory mechanisms.

### *PTPN6* and *miR-200c/141* promoters interact via a DNA loop

**Figure 8:**
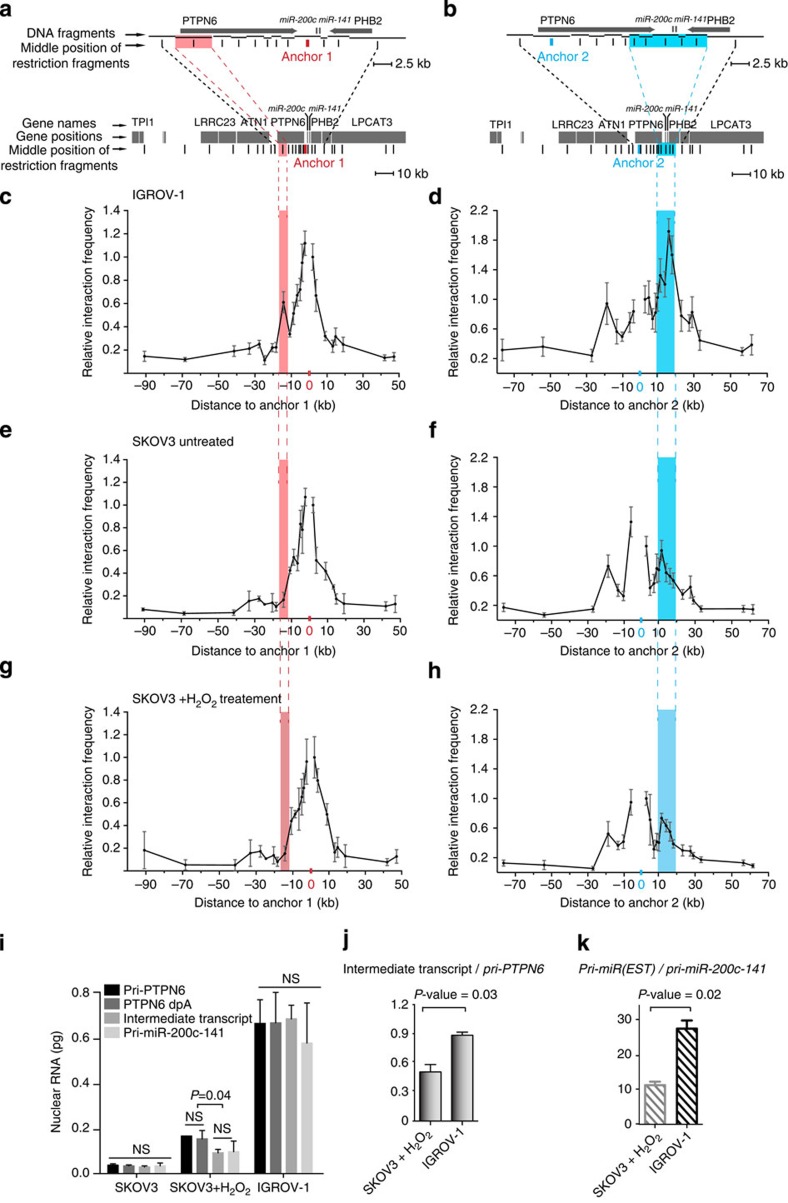
*PTPN6* and *miR-200c/141* promoters interact with each other through a DNA loop. (**a**,**b**) Schematic representations of *miR-200c/141* genomic locus, DNA fragments generated by chromatin digestion and anchors 1 (**a**) and 2 (**b**) used for 3C experiments. On the bottom part, genes surrounding *miR-200c/141*-coding region are shown as grey boxes. Each vertical line, indicated beneath boxes, corresponds to the middle position of each restriction fragment analysed. The upper parts of the schemas are high magnification of *PTPN6-miR-200c-141-PHB2* genomic locus. Black arrows indicate the sense of *PTPN6* and *PHB2* transcription. DNA restriction fragments, generated by chromatin digestion using *Nco*I restriction enzyme, are indicated by horizontal lines. Each vertical line, beneath the fragments, shows the middle position of each restriction fragment analysed. Are also indicated the positions of anchors 1 and 2 in *miR-200c/141* promoter (**a**) and *PTPN6* promoter (**b**), respectively. The red and blue shaded areas correspond to the genomic regions that interact with anchors 1 and 2, respectively. (**c**,**d**) 3C experiments from IGROV-1 ovarian cancer cells. The curves represent the relative interaction frequency of each DNA fragment with anchors 1 (**c**) and 2 (**d**), respectively. *X* axis represents the genomic distances from the anchor. Values are means±s.e.m. *n*=4 independent experiments. (**e**,**f**) 3C experiments from SKOV3 ovarian cancer cells. (**g**,**h**) 3C experiments from SKOV3 cell line exposed during 3 h to H_2_O_2_ treatment. (**i**) Quantity of each RNA entity detected along *PTPN6* and *miR-200c-141* genomic locus (pri-PTPN6, PTPN6dpA, intermediate and pri-miR-200c-141 primary transcripts), assessed by qPCR using cDNA from nuclear RNA of untreated SKOV3 cells, H_2_O_2_-treated SKOV3 cells and in IGROV-1 cells, as indicated. Quantifications were assessed using the standard curves shown in (Supplementary Fig.6a). Quantities are expressed in picogram (pg). (**j**,**k**) Ratios of quantities of Intermediate transcript versus *pri-PTPN6* primary transcript and *pri-miR(EST)* versus *pri-miR-200c-141* transcript in SKOV3+H_2_O_2_ and IGROV-1 cells. Data are means of±s.e.m. *n*=6 independent experiments per cell line. *P*-values are from Student's *t*-test. NS, not significant.

We finally wondered how their promoters of the *PTPN6* and *miR-200c/141* genes could be so finely co-regulated. We hypothesized that co-transcription of the two genes could result from a physical interaction between their promoters, thus facilitating regulation by common molecules, such as epigenetic regulators. To test this hypothesis, we performed chromosome conformation capture (3C) experiments (Fig. 8) that quantifies interaction frequency between genomic regions with high sensitivity for short-range chromatin interaction^54^. We first performed 3C experiments on the IGROV-1 ovarian cancer cell line expressing high levels of *PTPN6* and *miR-200c/141* transcripts (Fig. 8c,d; see Supplementary Fig. 1d and Supplementary Fig. 6c for comparative quantification of *PTPN6* and *miR-200c-141* primary transcripts in IGROV-1 and SKOV3 cell lines). Interestingly, we uncovered an interaction between *PTPN6*- and *miR-200c/141*-specific promoters, using a primer anchor (anchor 1) located at the *miR-200c/141* promoter region (Fig. 8c). The DNA loop detected using the anchor 1 brings together the 5′-end sequences of *miR-200c/141* and the *PTPN6* promoter regions. This interaction was further validated by the use of a reciprocal primer (anchor 2) located in the *PTPN6* promoter (Fig. 8d). Using anchor 2, we observed that *PTPN6* promoter not only interacts with *miR-200c/141* promoter, but also with a large genomic locus of ∼10 kilobases in length. This locus includes the *PTPN6* 3′-extremity, the *miR-200c/141* promoter region and a region downstream from the miRNAs that reaches the 3′-end of the *PHB2* gene (Fig. 8d). Thus, *PTPN6* gene is organized in a 3D gene-loop-like structure, which includes the *PTPN6* promoter and its 3′-extremity but also the *miR-200c/141* locus. In SKOV3 cells, characterized by low levels of *pri-PTPN6* and *pri-miR-200c/141* transcripts, the physical interaction between the two promoters was undetectable with the primer anchor 1 (Fig. 8e). Similarly, using the primer anchor 2, the interaction frequency between *PTPN6* and *miR-141/200c* promoters was close to background (Fig. 8f). Although the *PTPN6* intragenic-loop (bringing together *PTPN6* 5′- and 3′-ends) was still detected, it did not include the large downstream region containing *the miR-200c/141* gene (Fig. 8f). Moreover, although detectable, the interaction between *PTPN6* 5′- and 3′-ends was lower in SKOV3 than in IGROV-1 cells (Fig. 8d,f). This was expected as intragenic DNA loops have already been associated with transcriptional activity^50^,^55^. Altogether, these observations suggest that high-level expression of both *PTPN6* and *miR-200c/141* genes is associated with the formation of a 3D DNA loop associating their respective promoters. Still, the upregulation of both *PTPN6* and *pri-miR-200c-141* transcription by H_2_O_2_ treatment in SKOV3 cells was not enough to induce a detectable change in the 3D conformation (Fig. 8g,h).

### DNA loop is associated with increased read-through

**Figure 9:**
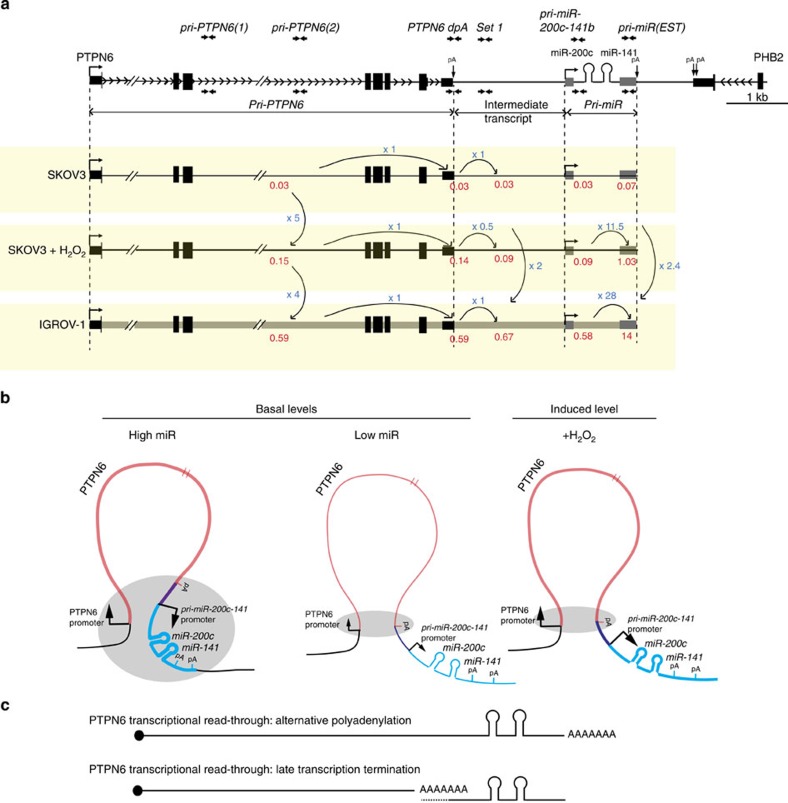
Model: miR-200c and miR-141 are co-transcribed with *PTPN6* by two complementary mechanisms, intergenic DNA-looping and transcriptional read-through. (**a**) Schematic representation of the *PTPN6*-*miR-200c/141* genomic locus and results obtained by quantitative analyses of the different primary transcripts detected along the locus, including *pri-PTPN6* primary transcript, Intermediate transcript, *pri-miR-200c-141* primary transcript and pri-miR(EST) exonic isoform, corresponding to previously described *ESTs*. Double arrows represent the primers used for detection of the transcripts. Quantities (in picograms) of each RNA entity detected along *PTPN6* and *miR-200c-141* genomic locus are shown in red. Fold changes are expressed in blue. (**b**) The short-range intergenic DNA-looping formed by the physical interaction between *PTPN6* and *miR-200c/141* promoters is associated with the transcription rate of the two genes. The grey-shaded ovals indicate the genomic regions, which have been defined as interacting with each other, according to 3C experiments. In cells characterized by high basal levels of *PTPN6* and *miR-200c/141* transcription, the *PTPN6* promoter can interact with a large region including the 3′-end of the *PTPN6* gene, the *miR-200c/141* promoter and downstream sequences. In cells expressing low basal levels of the two genes, the interaction is restricted to the 3′-end of *PTPN6* and does not include *miR-200c/141*. This conformation is not altered by the increased transcription upon oxidative stress (+H_2_O_2_), suggesting the existence of another mechanism is responsible for the co-expression of *miR-200c/141* and *PTPN6* following oxidative stress. (**c**) The transcriptional co-regulation between *PTPN6* and *miR-200c/141* can also be mediated by a transcriptional read-through of the *PTPN6* gene. This is highlighted by the intermediate transcripts, linking *PTPN6* to *miR-200c/141*, we identified. These intermediate transcripts can result from alternative polyadenylation or late termination of transcription by RNA-polymerase II, as schematically represented.

Finally, we investigated whether the DNA loop observed between the *PTPN6* and *miR-200c/141* promoters in IGROV-1 cells could favour the read-through from the *PTPN6* gene. To do so, we compared *PTPN6* primary transcript levels with that of the intermediate transcript in IGROV-1 and SKOV3 cells upon stress (Fig. 8i,j and Supplementary Fig. 5c for RT-negative controls). The ratio between intermediate transcript and *PTPN6* primary transcript levels was of 1 in IGROV-1 cell line, whereas of 0.56 in SKOV3 upon stress, indicating that the DNA loop is associated with increased read-through (Fig. 8j). In both SKOV3 and IGROV-1 cells, the levels of the different transcripts detected along the intermediate region and the *pri*-*miR-200c-141* genomic locus were equivalent among them (Fig. 8i). This means that independently of *PTPN6* transcription initiation rate and percentage of bypass, each molecule of the *PTPN6* bypassed transcript corresponds to one molecule of *pri-miR-200c-141* primary transcript, indicating a major role of the read-through. We also analysed the different quantities of the *pri-miR-200c-141* primary transcript (intronic isoform) with this one of the exonic isoform, corresponding to previously described *ESTs* (referred to as *pri-miR(EST),* see Fig. 5a for genomic localization). Globally, the quantity detected of *pri-miR(EST)* was at least tenfold higher than *pri-miR-200c-141* primary transcript (intronic isoform), most probably because *pri-miR(EST)* is stabilized and/or submitted to additive regulation. Here again, we observed that the differential expression between the two cell lines was maintained and 2.4-times higher in IGROV-1 than in SKOV3+H_2_O_2_ cells (Fig. 8k). Taken as a whole, our work shows that the 3D DNA loop linking *PTPN6-* and *miR-200c/141*-specific promoters contributes, with the transcriptional bypass of the *PTPN6* polyadenylation site, to the fine co-regulation of these two transcript units (Fig. 9).

## Discussion

We provide here evidence that intergenic miRNAs can be regulated through a transcriptional read-through and a 3D chromatin interaction between different promoters. The miR-200 family members exhibit key roles in oxidative stress response and ovarian tumorigenesis, oxidative stress being also an important feature of HGSOC^2^,^4^,^5^,^7^,^9^,^10^,^32^. Understanding the mechanisms involved in their regulation is an important question to highlight new insights of their functions in these patho-physiological conditions, especially in the characterization of ovarian cancer molecular subgroups. Here, we demonstrate that the transcription rate of the *miR-200c/141* transcription unit is a crucial step in determining mature miRNAs levels within cells, both at basal state and upon stress. Moreover, we unravel two original and complementary mechanisms by which the transcription of *miR-200c/141* is regulated: a transcriptional read-through of an upstream gene, *PTPN6,* and a transcription co-regulation mediated through a 3D DNA loop linking the two closely located *PTPN6* and *miR-200c/141* promoters.

Data from the last decade demonstrated that chromatin conformation is not randomly determined and has important roles in gene regulation. Studies in yeast have shown that DNA loops linking promoter and terminator regions of the same gene are important for transcriptional memory and directionality^56^. Examples of such 3D DNA conformation have also been described in humans and were associated with transcriptional repression^56^,^57^,^58^. Our data uncover a new example of such short-range chromatin interaction in human cells that can participate in miRNA regulation. Indeed, we highlight here a chromatin interaction that brings close together the promoters of *PTPN6* and *miR-200c/141* genes. Importantly, the chromatin loop we discovered between these two transcription units (*PTPN6* and *miR-200c/141*) does not correspond to the gene-loops previously described between the promoter and terminator regions within the same gene^58^,^59^,^60^. In contrast to these intragenic DNA loops, the chromatin loop we uncovered extends from the *PTPN6* promoter to a region far downstream of the *PTPN6* 3′-end, reaching the miR-200c/141 promoter and spanning at least 10 kilobases. Interestingly, we also detected that the promoter region of the *ATN1*-encoding gene, located upstream *PTPN6* may also interact with *PTPN6* and be another partner in the fine regulation of this genomic locus. The interaction between 5′-end of *PTPN6* and *miR* promoter could facilitate the RNA polymerase II recycling from the *PTPN6* 3′-end to its start site, as well as from the *pri-miR-200c-141* 3′-end to the *PTPN6* start site. Given the strict correlation of transcription between these two genes, we propose that the physical association of the two promoters plays a positive role in their co-regulation. Consistent with this idea, the formation of this 3D DNA loop is linked to the expression rates of the two genes and the proportion of read-through from the *PTPN6* gene. Although the 3D DNA structure is formed in cells with high basal levels of expression of the two genes, it remains undetected in cells with a low basal expression rate. Accordingly, the levels of DNA methylation and active histone marks, such as H3K9Ac and H3K4me3, are tightly equivalent between *PTPN6* and miR-200c/141 promoters. Some of the epigenetic silencing marks could be reversible and removed by oxidative stress. Our results are in agreement with previous studies showing that in breast, lung, colon, bladder and prostate cancer cells, miR-200c/141 levels are consistent with DNA methylation status and histone modifications detected in their promoter regions. We confirmed these results in ovarian cancer cells and extended them to the promoter of the upstream and co-regulated gene, *PTPN6*. Finally, we demonstrate here that the 3D DNA loop is associated with a 2.5-fold increase in transcription read-through from the upstream gene, *PTPN6*. It is important to note that given the physical interaction between the two promoters that we demonstrated here, the crosslink between the promoters could also contribute to the ChIP experiments results. Nevertheless, our results are consistent with the fact that both genes could be under equivalent epigenetic regulation, which is in complete agreement with the coordinated epigenetic and transcriptional regulation of these genes.

In addition to the DNA loop, our data unravel the read-through from the *PTPN6* gene, as another original mechanism linking *PTPN6* and *miR-200c/141* genes. Indeed, we demonstrate the existence of an intermediate transcript extending from the 3′-end of the *PTPN6* gene to the *miR-200c/141* transcription unit. It is mediated through the transcriptional bypass of the usual *PTPN6* polyA site, allowing the RNA polymerase II to reach the downstream *miR-200c/141* transcription unit. We identified two downstream APA sites used for the termination of the *pri-miR-200c-141* primary transcript. The first corresponds to the already established ESTs, located downstream of the miR-200c/141 genomic sequences. The second APA site, located very close to the *PHB2* polyadenylation signal, is conserved between human and mouse species and had not been reported previously (Supplementary Fig. 5). One should note, however, that the polyadenylation of the *PTPN6* transcripts at these APA sites is not necessarily required for the production of the miRNAs in the same round of *PTPN6* transcription. Indeed, miRNAs can be processed by Drosha co-transcriptionally and a late termination of RNA Pol II polymerization downstream from the *PTPN6* 3′-end can be sufficient for the transcription of the downstream miR-200c/141, even when *PTPN6* mRNA is polyadenylated at its usual site. Such regulation of intergenic miRNAs through upstream transcription has been postulated by Proudfoot and colleagues^61^. We give here, for the first time, evidence of this type of miRNA transcription.

We have investigated to which extent each of these two mechanisms (DNA loop versus *PTPN6* read-through) contributes to *miR-200c/141* primary transcription. The *pri-miR-200c-141* levels being the result of both *PTPN6* read-through and independent transcription initiation, we have carefully assessed the quantity of the different primary transcripts detected along *PTPN6* and *miR-200c/141* genomic locus. We observed that the read-through from the *PTPN6* gene is a dominant mechanism in ovarian cancer cells, each molecule of the *pri-miR-200c-141* primary transcript corresponding to one molecule of bypassed *PTPN6* polyadenylation signal. Thus, the transcriptional *PTPN6* read-through could be the major mechanism explaining the strikingly similar kinetics of *PTPN6* and *pri-miR-200c-141* upregulation following stress. Ideally, to demonstrate the impact of the *PTPN6* gene on *pri-miR-200c-141* transcription, a homozygous genetic knock-out model of the *PTPN6* gene would be required to definitely prove the role of *PTPN6* transcription on *miR-200c/141* regulation. A natural mutated mouse model for *Ptpn6* exists, the motheaten mice, but as the *Ptpn6* transcription is not impaired in these mice, they are not appropriate to investigate this question. We have thus evaluated if the read-through from the *PTPN6* gene can influence *miR-200c-141* transcription, by using the CRISPR/Cas9 technology. Deletion of *PTPN6* promoter demonstrated that *PTPN6* transcription is required for *miR-200c-141* primary transcription in SKOV3 cells. Unfortunately, *PTPN6* silencing is deleterious for the survival of IGROV-1 cells, characterized by high basal *PTPN6* expression rate. Being detected in cells with high expression levels, we can speculate that the 3D chromatin structure and subsequent high interaction frequency between the two promoters favours the co-regulated transcription of the two genes. The read-through molecule can reach the miRNAs locus, as demonstrated previously, or also favour the start of the polymerization from the miR-200c/141 TSS. In contrast, in cell lines that exhibit low basal expression rate of *PTPN6* and *miR-200c/141*, such as SKOV3, the interaction frequency between the two promoters is low. Interestingly, our data suggest also that the 3D DNA loop is not only associated with a high rate of transcription initiation but also of read-through from the *PTPN6* gene.

The so finely regulated co-expression of *PTPN6* and *miR-200c/141* suggest a functional relationship between the two genes. The levels of the miR-200 family members are reproducibly upregulated upon oxidative stress conditions in various cell lines and participate in stress response. Interestingly, the co-regulated gene *PTPN6* encodes for a Protein Tyrosine Phosphatase that is itself implicated in anti-oxidant defense^62^. Importantly, in HGSOC, the oxidative stress signature has a prognostic value and the miR-200s are significantly upregulated, as shown by several independent studies^2^,^4^,^5^,^7^,^32^. In that sense, our data on the mechanism of their regulation give another additive insight into the different ovarian transcriptomic subgroups defined by the miR-200/141. In complete agreement with our results, *PTPN6* is also generally overexpressed in ovarian cancer cells and in HGSOC^63^,^64^,^65^. PTPN6 and miR-200s play a physiological role in oxidative stress regulation and interestingly, they have both a negative impact on the p38 pathway. Indeed, in cells depleted for *PTPN6*, the levels of phosphorylated p38 are increased^66^. The activation of this key redox sensor that is also directly targeted by miR-14/200a (ref. 7) is rescued by a treatment with an antioxidant^66^. Finally, the 3C experiment also revealed that the promoter of the *ATN1* gene, located upstream *PTPN6*, could interact with *PTPN6* 5′-end. As ATN1 is involved in OXPHOS regulation and mitochondrial disorders^67^, this observation may represent interesting new clues for the global regulation of this genomic locus by oxidative stress, and for its role in this process. These results are complementary to previous studies^68^ showing a reciprocal interaction between miRNA and their regulatory transcription factor. Indeed, one of the largest known mammalian miRNA-containing cluster, Gtl2-Dio3, is regulated by MEF2A, a key transcription factor involved in skeletal muscle. Reciprocally, these miRNA directly target the secreted Fizzled-related protein 2, an inhibitor of the wingless-type (WNT) pathway. These observations are similar to our own findings showing the co-regulation of *PTPN6* and *miR-200c/141* by oxidative stress, the two exerting a role in oxidative stress response. Altogether, these data give complementary insights of the interplay between miRNA, their regulatory elements and the processes in which they are involved. The kind of transcriptional regulation we describe here are probably not restricted to these miRNAs and other examples of such mechanisms are likely to be discovered in the future.

## Methods

### Cell culture and treatments

All cells lines were cultured in DMEM (Invitrogen, # 41966) supplemented with 10% fetal bovine serum (except for immortalized mouse fibroblasts, which were maintained in 7% fetal bovine serum) at 37 °C and 5% CO_2_ atmosphere. Cells were treated with hydrogen peroxide, H_2_O_2_ (400 μM), except WI38 treated with 200 μM H_2_O_2_, for different periods of time, as indicated on each figure (up to 8 h). H_2_O_2_ was purchased from Sigma (#H-1009) and dilutions in H_2_O were prepared just before use. Mouse fibroblast cells were submitted for 3 h to various stresses increasing ROS levels: 50 μM menadine sodium bisulfite (Sigma, #M2518), 200 μM Arsenic (Sigma, #35000), 300 mM Sorbitol (Sigma, #85529). To inhibit transcription, cells were treated with 5 μM actinomycin D (Sigma, #A9415) during 4 h. The DNA-demethylating agent 5-Aza-2′-deoxycytidine (Sigma, #A3656) was diluted in dimethylsulphoxide and added to the cells at a final concentration of 10 μM. Medium was changed after 48 h and cells were treated for 96 h before lysis.

### RNA extraction

Total RNA isolation was performed using miRNEasy Kit from (Qiagen, #217004) according to the manufacturer's instructions. RNA concentrations were determined with a NanoDrop apparatus (NanoDrop Technologies, Inc.), and RNA integrity was verified using Agilent RNA 6000 Nano Kit (Agilent, #5067–1511) and apparatus. Before complementary DNA synthesis, RNA were treated with DNaseI, RNase free (Thermo Scientific, #EN0525) in reaction buffer with MgCl_2_ according to the manufacturer's protocol.

### Nuclear RNA extraction

Cells were resuspended in nuclear extraction buffer (Tris 10 mM pH 7.5; 10 mM NaCl; 5 mM MgCl_2_; 0.5% NP-40), incubated at 4 °C for 5 min and spin down 5 min at 500*g*. The cytosolic fraction was discarded. The pellet, corresponding to the nuclear fraction, was washed twice in the nuclear extraction buffer, resuspended in Trizol and passed through a 20-G needle five times. RNA extraction was done with mi-RNeasy kit (QIAGEN #217004) and RNA concentrations were determined with a NanoDrop apparatus (NanoDrop Technologies, Inc.). Before cDNA synthesis, nuclear RNA were treated with DNaseI, RNase free (Thermo Scientific #EN0525) in reaction buffer with MgCl_2_ according to the manufacturer's protocol.

### Real-time reverse transcription (RT)–PCR

Primary transcripts and mRNAs levels were assessed using SYBR qRT–PCR assay. Reverse transcriptase reactions were performed using 1 μg of DNAse-treated total RNA per sample and iScript RT Kit (Bio-Rad, #170–8897) or SuperScript III Reverse Transcriptase with oligo(dT) and random primers (Invitrogen, #180 80–051). SYBR Power master mix (Applied Biosystems, #4367659) was used for qPCR with primers at 300 nM final concentration each. *GAPDH, U6 snRNA* and *CYCLOPHYLIN B* were used as loading controls. TaqMan qRT–PCR assay was used for detection of mature miRNAs. Reagents, primers and probes were obtained from Applied Biosystems. RT reactions and real-time qPCR were performed according to manufacturer protocols from 50 ng of RNA per sample. Primers and probes are specific for each miRNA and are designed by the manufacturer. U6 snRNA and miR-16 were used as loading controls. qPCR reactions were performed in a Chromo4 apparatus (Bio-Rad). Relative expression was calculated using the comparative cycle threshold method (2^ΔΔCt^) and the Opticom Monitor 3 software. qPCR primers were designed using PrimerQuest (Integrated DNA Technologies) and were purchased from Sigma. The efficiency of each couple of primer used for qPCR was verified using serial dilutions of the template. When comparing transcript levels between different couples of primers, normalization using standard DNA was performed. Normalization was done using control DNA template obtained from the RP11-8J11 BAC clone (bacpac.chori.org), containing *PTPN6* and miR-200c/141 genomic region. For each primer pair, quantification was performed using the coefficients from the standard curve calculated using the control template dilutions measured in the same qPCR plate. Schematic representation of the genomic positions of the primers is indicated in Fig. 5a and sequences are given in Supplementary Table 2.

RT–PCR reactions using *Set 2* and *Set 3* primers were performed on cDNA from cells and HGSOC using Phusion DNA Polymerase (Thermo Scientific, # F-530) according to the manufacturer's instructions and with the GC buffer. During the first ten cycles of the PCR reactions, the annealing temperature increased 0.5 °C every cycle, from 60° to 65 °C and was maintained at 65 °C for the following cycles. The reactions were stopped after either 25, 30, 35 or 40 cycles. The PCR products were loaded on 0.8% agarose gel then transferred onto Hybond N+ membrane (GE Healthcare, # RPN203B). Membranes were hybridized with γ-ATP ^32^P-labelled primers (5′-TGACCCTGTATATAGCCCAGCCA-3′ and 5′-AGCAAACAAAGCCTGGGAGAGAGA-3′) in ULTRAhyb Ultrasensitive Hybridization Buffer (Ambion, # AM8669). Signals were detected with Typhoon and quantified with Multi Gauge software. All primer sequences used for PCR and qPCR are given in Supplementary Table 2. Amplified bands were purified, cloned in TA-vector (Invitrogen#45.0030) and submitted to sequencing in order to verify that they corresponded to the expected intermediate transcript sequence.

### CRISPR/Cas9 experiment

Target-specific CRISPR guide RNAs (sgRNA) were designed by GeneCoppeia to have limited off-targets. Corresponding primers were subcloned into the All-in-one sgRNA plasmid (GeneCopoeia, #CS-HCP000300-2-CG01-1-LR). The sequences flanking *PTPN6* promoter region targeted by the CRISPR guide RNAs are: sgRNA1: 5′-GATAACGCCTGCAACGACAT-3′; sgRNA2: 5′-GCCTGCCACCCACGGTAGAC-3′. Twenty-four hours before transfection, 5 × 10^5^ SKOV3 cells were seeded into six-well plates. Cells were transfected using Cell Avalanche Transfection Reagent (EZ-Biosystems #EZT-IGRO-1), provided by the company for enhancing transfection efficiency in IGROV-1 Ovarian cancer cells, but which also improved significantly the transfection efficiency in SKOV3 cell line. Three micrograms of sgRNA-recombined vector (containing the sgRNA1 and sgRNA2) were transfected using the manufacturer's protocol. Twenty-four hours post transfection, the cells were transiently selected (for 24 h) with hygromycine B Gold (InvivoGen, #ant-hg-1) in order to eliminate non-transfected cells. Transfection protocol was repeated four times on total cell populations. Sixty-three individual clones were next isolated by limited dilution and analysed at genomic level. PCR- and qPCR-based genotyping methods were used to identify the clones in which the genomic deletion occurs and to quantify the DNA copy number of the targeted region relative to control regions, measuring the efficiency of CRISPR deletion as creating allelic deletions, SKOV3 cells being tetraploid. Sequences of the primers used for the genotyping were designed either inside or outside the targeted region, as schematically represented in Fig. 6a. Sequences of the primers are given in Supplementary Table 2. Genomic DNA of the 60 individual clones was purified with DNAeasy blood and tissue kit (Qiagen, #69504) using the manufacturer's instructions. qPCR was performed with 50 ng of genomic DNA in duplicate using SYBR green master mix (Roche) on a Chromo4 apparatus (Bio-Rad). Relative levels of DNA copy number were calculated using the 2^ΔΔCt^ method with copy number normalized to glyceraldehyde-3-phosphate dehydrogenase (GAPDH) intron 1 (chromosome 12) as a control. Two individual clones exhibited a significant reduction of DNA copy number in the targeted region, with two- and three-recombined alleles among four, respectively. To obtain complete deletion of the four alleles, these two individual clones were submitted to two additive rounds of transfections and re-analysed at genomic level, using the same PCR- and qPCR-based genotyping method, as described above. Two clones referred to as SKOV3-Δ1 and SKOV3-Δ2 exhibited tetra-allelic recombination and deletion of the PTPN6 promoter-targeted region and were considered for further analyses. The amplified fragments from genotyping PCR and qPCR have been isolated, cloned and sequenced to verify that they correspond to the recombined *PTPN6* genomic locus after deletion.

### HGSOC cohorts

The Institut Curie cohort is described in ref. 7. Analyses based on the Curie cohort were approved by the Institutional Review Board and Ethics committee. Before inclusion in the study, patients were informed that their biological samples could be used for research purposes and that they had the right to refuse if they wished so. Analysis of tumour samples was performed according to the relevant national law on the protection of people taking part in biomedical research. Only samples enriched in at least 65–70% epithelial cells have been considered in our study. miRNA and mRNA levels were assessed by qPCR using TaqMan and SYBR assays as described in ref. 7. Microarray data are accessible in the Gene Expression Omibus under the accession number GSE26193. All the data from the TCGA cohort was obtained from the TCGA Data Portal (https://tcga-data.nci.nih.gov/tcga/). Patient characteristics and clinical features of the two cohorts analysed in our study are listed in Supplementary Table 1, in order to facilitate their comparison. TCGA data are provided from tumour samples with at least 60% of enrichment in epithelial tumour cells, as mentioned in the Supplementary Information in ref. 51.

### Copy number analyses in ovarian cancer samples

For the Curie cohort, SNP 100K data were analysed using Partek Software. Raw data were imported into Partek using default parameters and the Partek baseline for copy number calculation, followed by a GC-adjustment. The segmentation parameters used were 15 markers, a signal-to-noise ratio of 0.3 and a *P*-value of 0.001. The threshold used for defining amplified/deleted patients in the *PTPN6* and *miR-200c/141* region was ±0.3. For the TCGA cohort, copy number data were downloaded from: https://tcga-data.nci.nih.gov/tcga/ (Level 3 data, MSKCC 1x1M-G447A). Data were imported and analysed using R version 2.12.2. Segments defined in TCGA data were used to classify unchanged, amplified or deleted patients in the region of interest. The classification was made according to segment mean values in a window of 150 kb in the chromosome 12p13 including *PTPN6* and *miR-200c/141*. Patients were considered as ‘Amplified' or ‘Deleted', if any segment mean value in this region was above +0.3 or below −0.3, respectively. ‘Unchanged' patients were those who exhibit neither amplifications nor deletions in the region of interest, according to this threshold.

### TCS analyses

TCS were computed as described in ref. 52. Briefly, from the microarray data, a list containing only one probeset per gene is made. Next, for each gene, the TCS is computed as the sum of the correlation coefficients (Spearman's rho coefficient) between the gene and its 2n neighbours (*n*=10). To define a significance threshold (*P*=0.001), TCS were computed using randomized positions for probesets and the TCS of the 1,000th quantile was used. For the TCS Maps, TCS values of each gene are plotted against the corresponding genomic position.

### NCI-60 and Sanger Cell Line Project data sets

For the NCI-60 panel, Agilent microarray data were obtained from CellMiner Database (http://discover.nci.nih.gov/cellminer/loadDownload.do) and miRNA qPCR data were obtained from the Israel lab http://dtp.nci.nih.gov/mtargets/download.html. For the Sanger Cell Line Project panel, microarray data were obtained from the Broad Institute website (http://www.broadinstitute.org/cgi-bin/cancer/publications/pub_paper.cgi?mode=view&paper_id=189). Affymetrix Plus 2.0 probeset 206687_s_at was used for PTPN6 levels. All the non-haematopoietic cell lines were included in the analyses.

### Methylated DNA Immunoprecipitation

MeDIP experiments were performed using MagMEDIP kit from Diagenode (#C02010020), according to the manufacturer's instructions. Briefly, genomic DNA was extracted from 3 to 10 million cells and sonicated using the Bioruptor apparatus (Diagenode) to obtain fragments of 100–600 bp in length (10 min at ‘low' power with cycles of 15 s ON and 15 s OFF). For each reaction, 1 μg DNA was used. Positive (methylated DNA) and negative (unmethylated DNA) controls were added to the reaction, in order to normalize for the immunoprecipitation (IP) efficiency. IP using anti-5-methylcytosine antibody and magnetic beads was performed overnight at 4 °C. After washing and elution, immunoprecipitated DNA was measured by qPCR and reported to input DNA.

### Chromatin immunoprecipitation

Cells were crosslinked for 15 min using formaldehyde (1% final concentration) (Sigma, #F8775) at room temperature. Next, they were harvested and nuclei were extracted, lysed and sonicated. IP was performed using magnetic beads (Invitrogen, #100-02D) and 5 μg of antibody for each condition. Anti-RNA Polymerase II antibody was purchased from Santa Cruz (sc-899) and histone antibodies were purchased from Abcam: H3K4me3 (ab8580), H3K9Ac (ab10812), H3 (ab1791). After reverse crosslink, ChIP enriched fragments were evaluated by qPCR and reported to input DNA levels.

### Chromosome conformation capture

3C experiments were performed based on protocols previously established^69^ with modifications. Fifty million cells were crosslinked with 2% formaldehyde for 10 min at room temperature and lysed in the following buffer: 10 mM Tris-Cl, pH 8.0; 10 mM NaCl; Igepal (NP-40), 0.2% (v/v) containing protease inhibitors. DNA was digested overnight with the restriction enzyme *Nco*I (New England BioLabs) in a buffer containing final concentration of 1% Triton X-100 and 0.1% SDS. The restriction enzyme was chosen in order to investigate the short-range chromatin interaction with a high-resolution capacity in our region of interest. Ligation in diluted conditions (total of 80 ml) was performed at 16 °C for 4 h followed by 30 min at room temperature using T4 ligase from Promega (# M1794). After overnight reverse crosslink at 65 °C with proteinase K (Roche, #03 115 836 001), DNA was extracted with phenol and chloroform and precipitated in ethanol. RNAse (Roche, #11 119 915 001) treatment was performed followed by supplementary phenol chloroform extractions and ethanol precipitation. For each experiment, purity of 3C template was assessed as described in ref. 69. Restriction efficiency at each *Nco*I site was calculated from non-ligated controls and was of 60–70% in average. Primer normalization was done using control template obtained from digestion and ligation of the RP11-8J11 BAC clone (bacpac.chori.org). For each primer pair, quantification was performed using the coefficients from the standard curve calculated using the control template dilutions measured in the same qPCR plate. For each fragment, a relative interaction frequency over the first 3′ fragment from the anchor was computed (by dividing the interaction frequency of a given fragment to this one of the closest 3′-fragment from the anchor) and plotted according to its distance to the anchor. All primers and modified probes were purchased from MGW Operon. Primer and anchor (probes) sequences are given in Supplementary Table 3.

### 3′ and 5′ RACE

RACE experiments were performed using the RLM-RACE kit from Ambion (#AM 1700) according to the manufacturer's instructions. The 5′ RACE protocol is designed to detect only capped mRNA. Primers used for RACE experiments are listed in Supplementary Table 2.

### ZEB1, ZEB2 or TP53 silencing

2 × 10^5^ SKOV3 and IGROV-1 cells were transfected the day after seeding with 10 nM of each siRNA using Dharmafect 1 transfection reagent (Dharmacon, T-2001) according to the supplier's recommendations. Forty-eight hours post transfection, SKOV3 cells were either untreated or treated with 400 μM H_2_O_2_ for 1 h before RNA extraction and purification using miRNeasy kit from Qiagen. mRNA levels were measured using SYBR RT–qPCR (see above) with specific primers. GAPDH was used for internal control of mRNA quantity. SKOV3 cells are *TP53* null and no expression of ZEB2 was detected in IGROV-1 cell line, thus SKOV3 and IGROV-1 were not silenced using *TP53-* and *ZEB2*-specific siRNA, respectively. siRNA used for transfection were: siZEB1 (smart pool from Dharmacon #L-006564); siZEB2 (smart pool from Dharmacon #L-006914); siTP53 (smart pool from Dharmacon #L-003329) and siCtl (Control; Dharmacon #D-001810-01). Primer sequences used for qPCR are given in Supplementary Table 2.

### Statistic analyses and databases

Data are generally reported as means±s.e.m. (or s.d. when specified), obtained from at least three independent experiments, using adapted statistical test, as mentioned. Correlation coefficients are indicated by *ρ* (rho) and correspond to Spearman's rank correlation coefficients. The genome sequence data were obtained from University of California at Santa Cruz (genome.ucsc.edu).

## Additional information

**How to cite this article:** Batista, L. *et al.* Regulation of miR-200c/141 expression by intergenic DNA-looping and transcriptional read-through. *Nat. Commun.* 7:8959 doi: 10.1038/ncomms9959 (2016).

## Supplementary Material

Supplementary Information Supplementary Figures 1-7 and Supplementary Tables 1-3
