# Supplementary material for: Regulation of miR-200c/141 expression by intergenic DNA-looping and transcriptional read-through
Source: Nat Commun. 2016 Jan 4;7:8959. doi: 10.1038/ncomms9959 (PMC4727242; doi:10.1038/ncomms9959)
Supplement: Supplementary Information — Supplementary Figures 1-7 and Supplementary Tables 1-3 [file ncomms9959-s1.pdf]

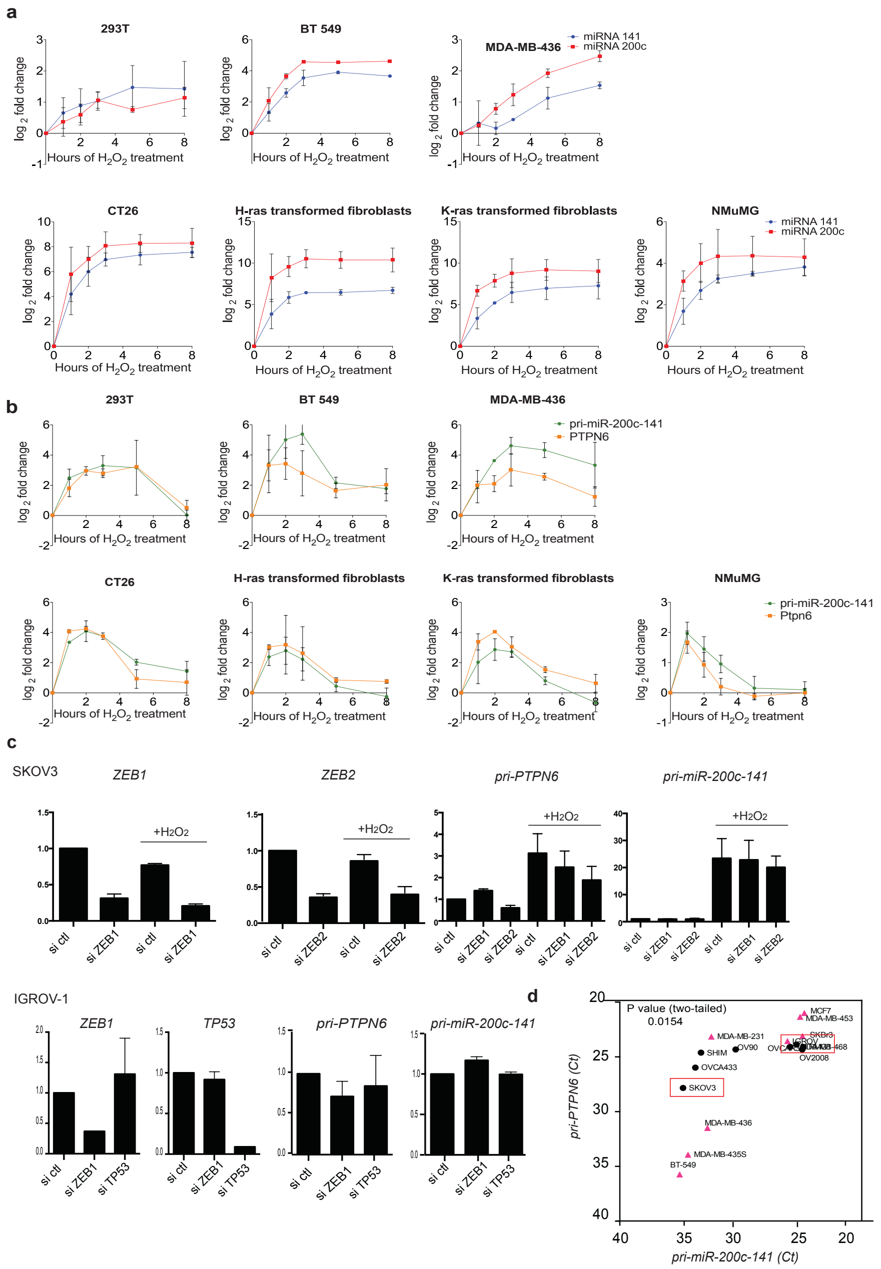

Supplementary Figure 1

(**a**,**b**) Kinetics of accumulation of mature miR-141 and miR-200c microRNAs (**a**), and *pri-miR-200c-141* and *pri-PTPN6* primary transcripts (**b**) following H_2O_2 treatment in human (293T Kidney cells, and BT549, MDA-MB-436 breast cancer cells) and mouse (CT26 colon carcinoma, Ha- and Ki-ras transformed fibroblasts, NMuMG epithelial) cell lines, as indicated. qRT-PCR data are means of fold changes (normalized to untreated and expressed as log_2) ± SEM. n ≥ 2 independent experiments per cell line. (**c**) Effect of ZEB1, ZEB2 or TP53 silencing on the levels of *pri-PTPN6* and *pri-miR-200c-141* primary transcripts*,* in (**Top**) SKOV3 cells either untreated (NT) or following 3 hours of H_2O_2 treatment (+H_2O_2) and in (**Bottom**) IGROV-1 cell line, as indicated. qRT-PCR data are shown as fold change ± SD normalized to control siRNA. *n* ≥ 2 independent experiments per cell line. SKOV3 cells are *TP53* null and no expression of ZEB2 was detected in IGROV-1 cell line, thus SKOV3 and IGROV-1 were not silenced using *TP53* and *ZEB2*-specific siRNA, respectively. (**d**) Correlation plot of cycle threshold of primary *PTPN6* and *pri-miR-200c-141* transcripts in ovarian (SKOV3, OVCA433, SHIN-3, OV90, OVCAR3, OV2008, RMG1, IGROV-1, listed from the lowest to the highest *PTPN6* and *pri-miR-200c-141* expression rate) and breast (BT549, MDA-MB-435, MDA-MB-436, MDA-MB-231, SKBR3, MDA-MB-453, MCF7) cancer cell lines. SKOV3 and IGROV-1 cell lines are highlighted by red boxes. In our work, the “High-“ and “Low-expressing” subgroups have been defined by the mean of expression of the two genes (*PTPN6* and *miR-200c/141*) in the cell lines considered.

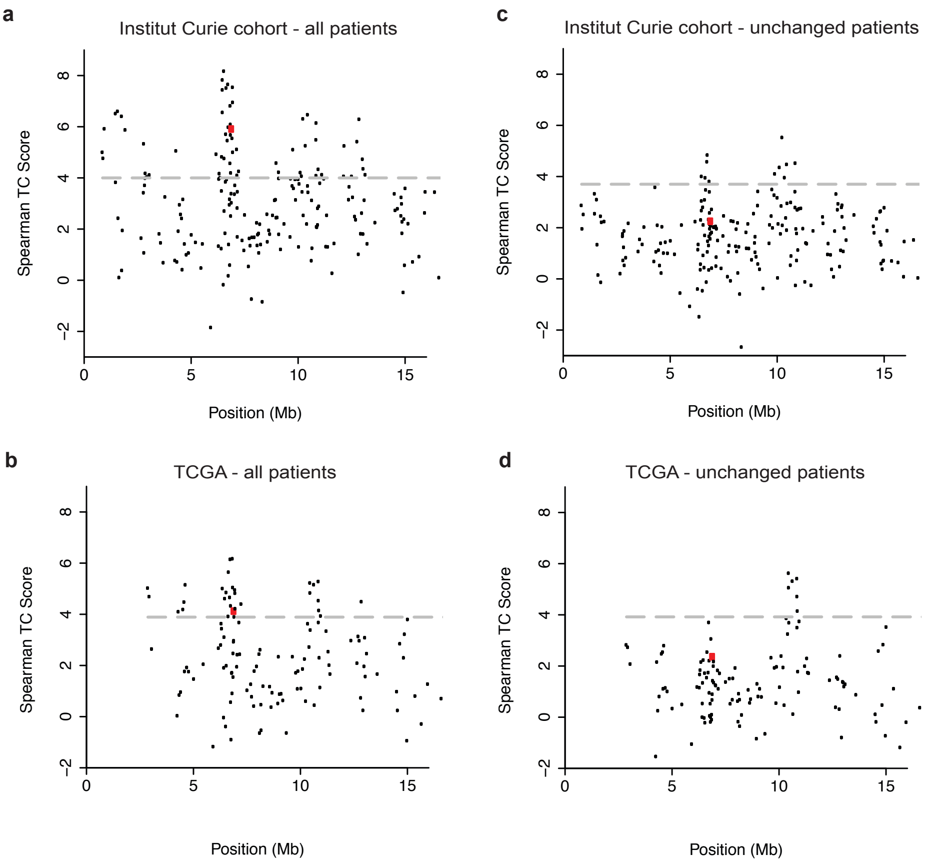

e Number of patients in each subgroup

	Institut Curie cohort	TCGA
Unchanged	51	320
Amplified/Deleted	53	231
Not Available	3	23
**All patients**	**107**	**574**

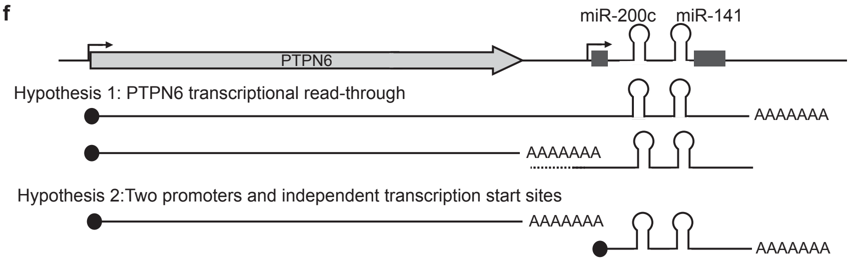

Supplementary Figure 2

(**a**,**b**) Transcription Correlation (TC) Score Maps in the chromosome 12p13 of ovarian adenocarcinomas from the Institut Curie (**a**) and TCGA (**b**) cohorts of patients. TC Score is defined as the sum of the Spearman correlation coefficients of a given gene with its 20 neighboring genes, as described previously in [85]. In each graph, the dashed horizontal line represents the threshold of significance (*p-value*=0.001) determined using randomized probesets (see Materials and Methods). The *PTPN6* gene is indicated in red. (**c**,**d**) Same as (**a**,**b**) considering only patients without copy number alteration in the *PTPN6-miR-200c/141* locus (referred to as “Unchanged” patients). (**e**) Number of patients studied for TCS analyses, in sub-groups classified according to the copy number status, “Unchanged”, “Amplified/deleted” or undetermined (NA), as indicated. (**f**) Schematic representation of two non-exclusive hypotheses explaining the correlation of transcription between *PTPN6* and its downstream miR-200c and miR-141. In the first hypothesis, *PTPN6* and *miR-200c/141* are transcribed together, through bypass of *PTPN6* usual polyadenylation site and transcriptional read-through. In the second hypothesis, *PTPN6* and *miR-200c/141* genes are transcribed by independent promoters and exhibit their own independent capped and polyadenylated primary transcripts.

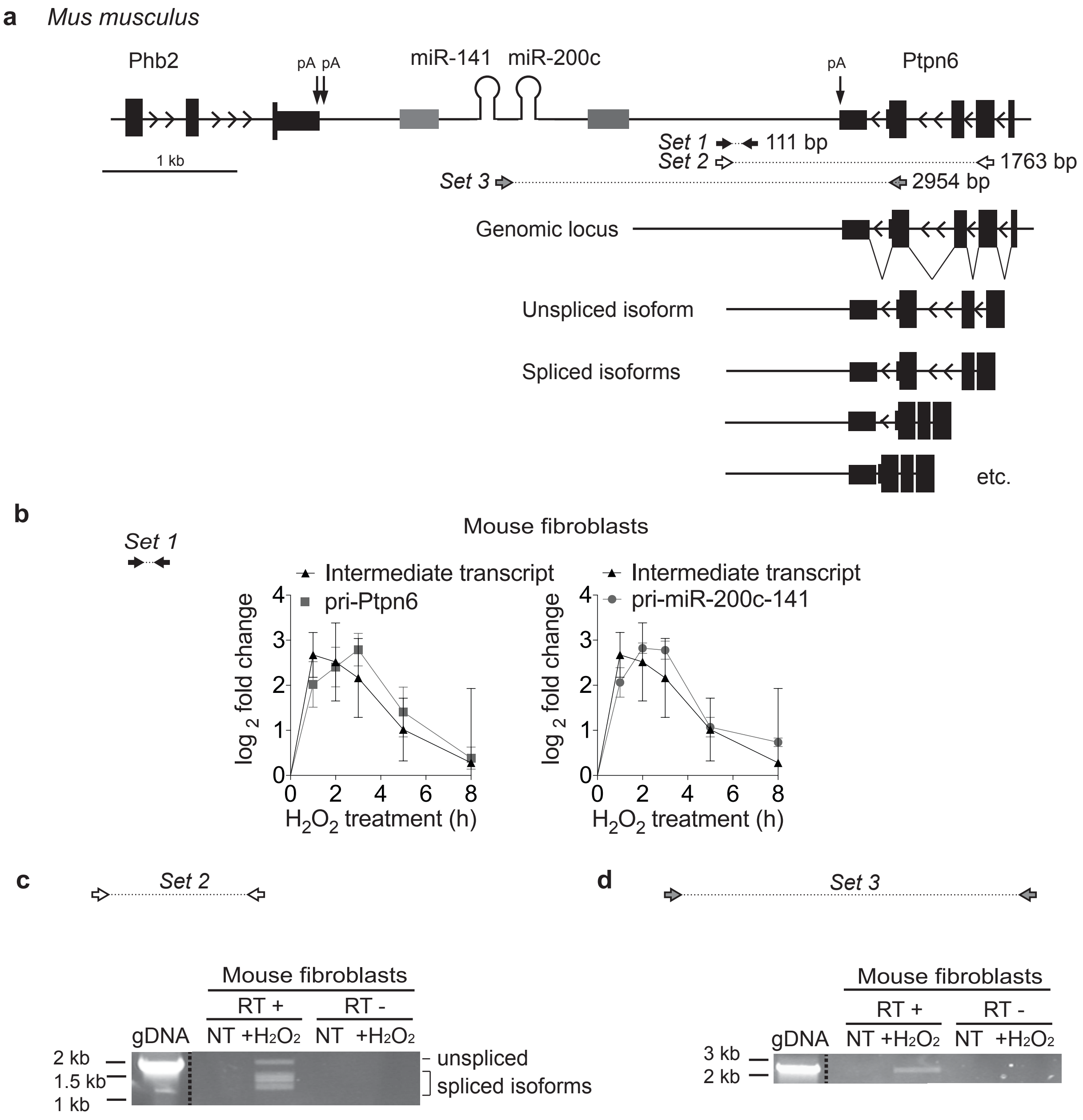

Supplementary Figure 3

(**a**) Schematic representation of the mouse genomic locus showing miR-200c/141 and their neighboring genes, *Ptpn6* and *Phb2 (*schema adapted from UCSC browser: https://genome.ucsc.edu). Black and grey boxes represent exons and ESTs, respectively. Polyadenylation sites (pA) are indicated. Double arrows represent the position and orientation of the primers (*Sets 1, 2*, *3*) used for the experiments. *Set 1* was used for qRT-PCR (**b**) and *Sets 2* and *3* for RT-PCR (**c,d**). Lengths of the amplified products are indicated in base pairs (bp). Sequences of the primers are given in **Supplementary Table 2**. Schematic representation of Spliced and Unspliced isoforms of the transcripts are indicated. (**b**) Kinetics of accumulation of the intermediate transcript detected using *Set 1* primers, upon H_2O_2 treatment in mouse fibroblasts, and compared to *pri-Ptpn6* (**left**) or *pri-miR-200c/141* (**right**) primary transcripts. qRT-PCR data are means of fold changes (normalized to untreated and expressed as log_2) ± SEM. n ≥ 3 independent experiments. (**c,d**) PCR reactions using *Set 2* (**c**) and *Set 3* (**d**) primers showing the existence of intermediate transcripts spanning from the 3'-end of the *Ptpn6* gene and reaching the miR-200c/141 locus. Representative amplifications using genomic DNA (gDNA) or cDNA from untreated (NT) or H_2O_2 treated (+H_2O_2) cells. RT- indicates control without the reverse transcriptase enzyme.

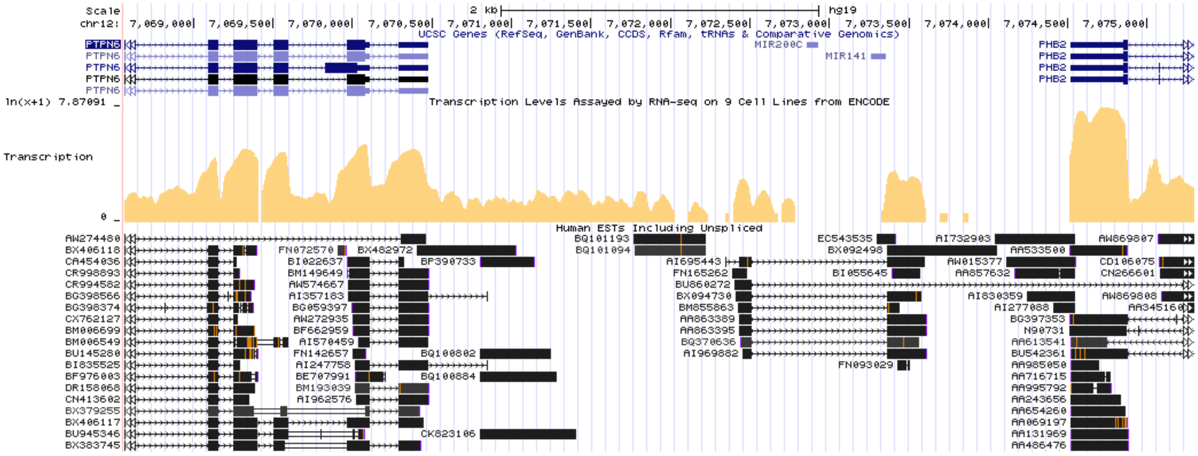

Supplementary Figure 4

Transcription levels assessed by RNA-seq data obtained from 9 cell lines from the ENCODE project. Data are exported from UCSC Genome Browser: http://genome.ucsc.edu/cgi-bin/hgTracks?db=hg19&position=chr12%3A7068567-7075296&hgsid=435585123_0m8g0nZab43PawLfJzAQuRQZkhOs and show *PTPN6* and miR-200c/141 genomic region in human being. Genomic positions, scale and genome version used (*hg19*) are indicated on the top. Horizontal arrows indicate the sense of transcription. Coding exons are indicated as blocks connected by horizontal lines representing introns, and arrowheads showing sense of transcription. Untranslated regions are presented by thinner blocks. In yellow are shown the transcription rate in H1-hESC cells at the locus, including unspliced RNAs. RNAseq data are presented with log scale (ln(x+1)), intronic expression being around 10 copy, and exonic expression being around 100 to 200 copies. The levels of expression of this intermediate transcript, while being much lower than mature (exonic) *PTPN6* mRNA -as expected- reached the same expression rate as the pri-*PTPN6* primary (intronic) transcript. Detailed information about the experimental procedures and data analysis are available on UCSC website: http://genome.ucsc.edu/cgi-bin/hgTrackUi?db=hg19&g=wgEncodeRegTxn ; and http://genome.ucsc.edu/cgi-bin/hgTrackUi?g=wgEncodeCaltechRnaSeq.

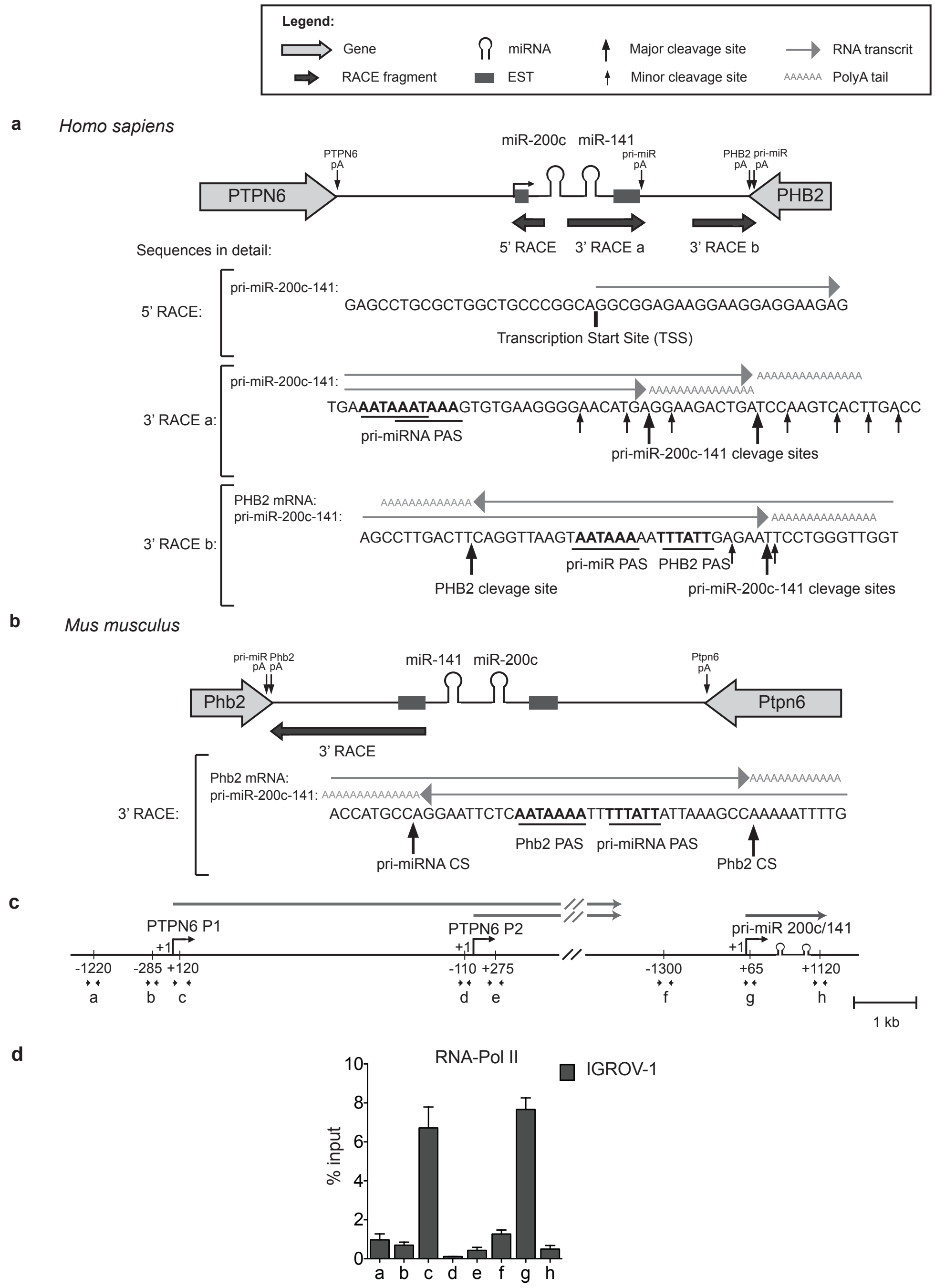

Supplementary Figure 5

Schematic representation of the characterization of the 5'- and 3-ends of the *pri-miR-200c-141* primary transcript, evaluated using 5' and 3' RACE in cells from human (**a**) and mouse (**b**) origins. The neighboring genes, *PTPN6* and *PHB2,* are shown and large gray arrows indicate the sense of their transcription. Primers used for 3' and 5' RACE and the corresponding transcript amplifications are represented on the bottom of each schema according to the legend. Polyadenylation sites (pA) of each gene are indicated. Detail of sequences and polyadenylation sites identified by RACE experiments are given on the bottom part. Polyadenylation signals (PAS) are underlined and cleavage sites (CS) indicated by arrows, smaller arrows represent less frequently observed or minor CS and larger arrows indicated the most frequent CS. **(c)** Schematic representation of *PTPN6* and *pri-miR-200c-141* genomic organization showing the localization of the primers (referred to as a-h) used for MEDIP and ChIP experiments. Relative position of the primers to each promoter (indicated for each gene as +1) is shown. Sequences are found in **Supplementary Table 2. (d)** ChIP experiments using a RNA-Polymerase II (RNA-Pol II)-specific antibody in IGROV-1 cells showing an enrichment of RNA-Pol II on the transcription initiation region of the PTPN6 and pri-miR-200c-141 genes (regions corresponding to primers c and g, as indicated in (**c**). Values are means ± SEM. n=3 independent experiments.

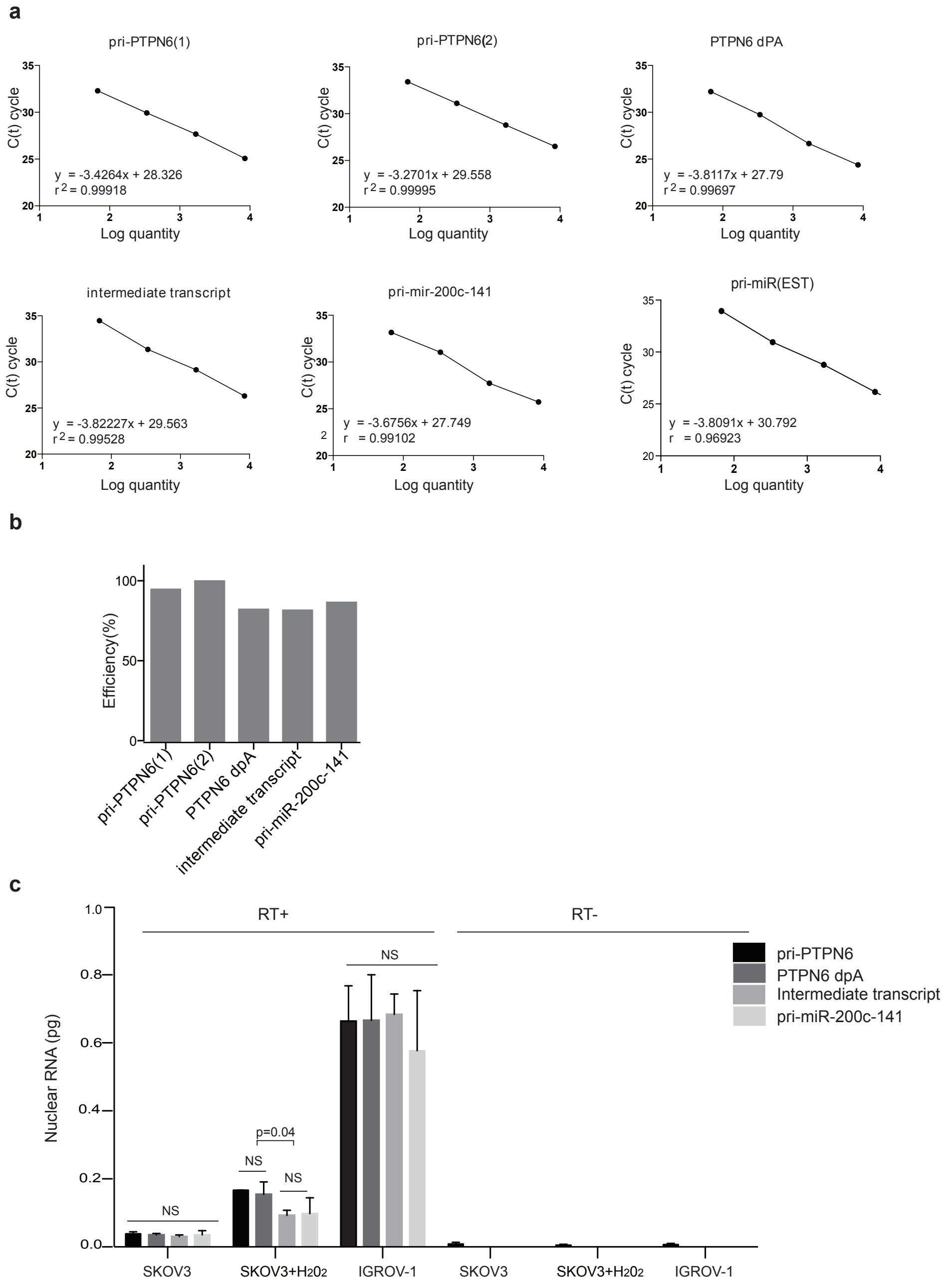

Supplementary Figure 6

(**a**) Standard curves obtained with the different couples of primers (as indicated) on serial dilutions of a BAC DNA, containing *PTPN6* genomic region. Schematic representation and sequences of the primers are given in in **Fig. 4a** and in **Supplementary Table 2**, respectively. In brief, pri-PTPN6(1) and pri-PTPN6(2) primers detect *pri-PTPN6* primary transcript, PTPN6dpA primers overlap with *PTPN6* polyadenylation signal, *Set 1* primers allow the detection of the Intermediate transcript and pri-miR-200c-141b primers detect *pri-miR-200c-141* primary transcript. (**b**) Efficiency of amplification obtained using the different couples of primers, as indicated. **(c)** Quantity of each RNA entity detected along *PTPN6* and *miR-200c-141* genomic locus, assessed by qPCR using cDNA from nuclear RNA of untreated (NT) or H_2O_2-treated SKOV3 cells and in IGROV-1 cells, as indicated. Quantifications were assessed using the standard curves shown in (**a**). RT- indicates controls without the reverse transcriptase enzyme. Only significant differences in each cell type are indicated.

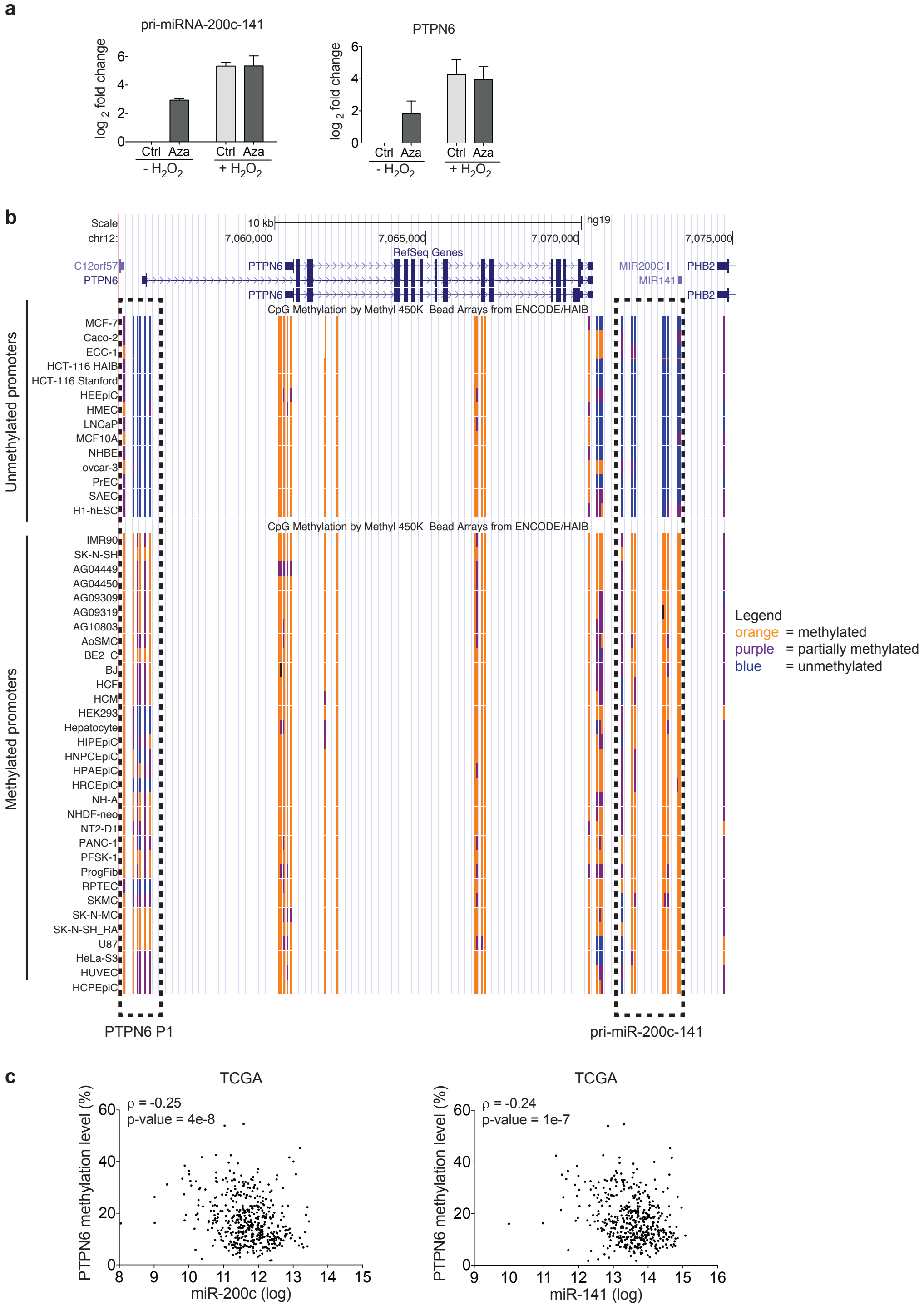

Supplementary Figure 7

(**a**) Effect on SKOV3 ovarian cancer cells of 96 hours of treatment with a DNA-demethylating agent, 5-Aza-2'-deoxycytidine (Aza), on the levels of *pri-miR-200c-141* (left) and *PTPN6* (right) primary transcripts under untreated conditions (-H_2O_2) or following 3 hours of H_2O_2 treatment (+H_2O_2). qRT-PCR data are shown as fold changes following Aza treatment (normalized to control (Ctrl) and expressed as log_2). n=3 independent experiments. (**b**) Methylation status of different cell lines (as indicated) from the ENCODE Project on the *miR-200c/141* and *PTPN6* promoters. The color code is as followed: orange represents highly methylated CpG islands, purple, partially methylated CpG islands and blue, unmethylated CpG islands. (**c**) Correlation plot showing that miR-200c (left) and miR-141 (right) expression levels are negatively correlated with DNA methylation rates in the PTPN6 P1 promoter assessed by the probe cg04956511 in the TCGA cohort.

		Institut Curie	TCGA
**Total number of tumors**		107	574
**Median age (range) - years**			
	**Median age**	58 years	59 years
	**Range**	31-86 years	27-87 years
**Histotype**			
	**Serous**	82 (76.5%)	
	**Endometrioïd**	8 (7.5%)	
	**Mucinous**	8 (7.5%)	
	**Clear cell**	6 (5.5%)	
	**Carcinosarcoma**	2 (2%)	
	**Brenner tumor**	1 (1%)	
**Figo substage**			
	**I**	21 (19.6%)	
	**II**	10 (9.3%)	24 (4.2%)
	**III**	59 (55.1%)	377 (65.7%)
	**IV**	17 (15.9%)	78 (13.6%)
	**NA**		95 (16.6%)
**Grade**			
	**1**	7 (6.5%)	
	**2**	34 (31.8%)	57 (9.9%)
	**3**	66 (61.7%)	415 (72.3%)
	**NA**		102 (17.8%)
**Surgery**			
	**Optimal**	38 (36%)	310 (54%)
	**Subptimal**	69 (64%)	264 (46%)
**Relapse**			
	**Yes**	80 (75%)	345 (60%)
	**No**	27 (25%)	136 (24%)
	**NA**		93 (16%)
**Genomic status at miR-200c/141 locus**			
	**Unchanged**	51 (48%)	320 (56%)
	**Amplified/Deleted**	53 (49%)	231 (40%)
	**NA**	3 (3%)	23 (4%)
**miRNA data**			
	**Available**	82	570
	**NA**	25	4
**Reference**			
		Mateescu et *al*. 2011	TCGA 2011

Supplementary Table 1

Patient characteristics and clinical features of the cohorts referred to as Curie and TCGA have been previously described in [36,84] and in https://tcga-data.nci.nih.gov/tcga/ (see also Methods). Characteristics are based on disease at diagnosis, including histological subtype, grade, stage and extent of residual disease post-surgery. Surgery was defined as optimal for patients with no macroscopic tumor residues visible after resection, and suboptimal for detectable tumor residues after surgery. Genomic status at miR-200c/141 locus was defined for each patient as described in the Method section. NA: Not available.

qPCR primer sequences

**MOUSE PRIMERS (5'->3')**	
pri-miR-200c-141 F(b)	GGTTGCCCACTGGAAGAACACAAT
pri-miR-200c-141 R(b)	TAGACAATCCCAAGGCCAAGGTCTG
pri-miR-200c-141b F(a)	AAGACGCTGGCTTCCGTTGTCTTT
pri-miR-200c-141b R(a)	AGAGCTTCAGCTCCTTGGGTTCTT
pri-Ptpn6 (1) F	GCAAATGTGAACAGACCACAGGCA
pri-Ptpn6 (1) R	TTGGATGGCCATTTGGGAAGAAGC
pri-Ptpn6 (2) F	CAATCAAGGCAAGGCCACCTCATT
pri-Ptpn (2) R	TTGCCATCTATGGCTCTGTGGCTA
Ptpn6 mature F	GCAAACTTTCCTGTCGCTGGTTGA
Ptpn6 mature R	TTCGGGAGATATGGCACTACCAGT
Pri-Phb2 1 F	ATGTCCTGTAGATCGCCACATCAC
Pri-Phb2 1 R	ACTTTCATCCTGTAGATTCAGCACAAGG
Set 1 F	TGTGTGGACTAATTCTGGCCTGGT
Set 1 R	TAACGCCCAGTGTGTTAAGGGTCA
Gapdh F	AGGTCGGTGTGAACGGATTTG
Gapdh R	TGTAGACCATGTAGTTGAGGTC
U6 F	TCGCTTCGGCAGCACATA
U6 R	ACGAATTTGCGTGTCATCCT
**HUMAN PRIMERS (5'->3')**	
pri-miR-200c-141 F(b)	CTGCCTTTACAGCTGCAGCAAGAT
pri-miR-200c-141 R(b)	TTTATTTCATGCTCCCAAGGCGGG
pri-miR-200c-141b F(a)	GTAAATCGGTGTGTGTCGCGGGTC
pri-miR-200c-141b R(a)	CCGAGTCCCTGGGGACACTTC
pri-PTPN6 (1) F	TCTCCGGCCCAAACAGAGATCATT
pri-PTPN6 (1) R	AAAGGAGCCATGGTGTCATGAGGA
pri-PTPN6 (2) F	TTACCAGCTGTGTGGTCTTGGACA
pri-PTPN6 (2) R	ATTCCAGCCACAACGATAGGGTCT
PTPN6 mature F	GGTGTCCACGGTAGCTTCC
PTPN6 mature R	ACAGGTCATAGAAATCCCCTGAG
pri-PHB2 1 F	ATCTTGGCAGCCTCGGCCTCA
pri-PHB2 1 R	AGCGGGCCCAATTCTTGGTAGAAA
ZEB1 F	TTCAAACCCATAGTGGTTGCT
ZEB1 R	TGGGAGATACCAAACCAACTG
ZEB2 F	CAAGAGGCGCAAACAAGC
ZEB2 R	GGTTGGCAATACCGTCATCC
TP53 F	AGGCGATCCACCTGTCTCA
TP53 R	CAGATGTGCTTGCAGAATGT
Set 1 F	AGCAAACAAAGCCTGGGAGAGAGA
Set 1 R	TGTATAGAAAGGGCGTTGCTGGGA
GAPDH F	GAAGGTGAAGGTCGGAGTC
GAPDH R	GAAGATGGTGATGGGATTTC
CYCLO B F	GCTAGATGGCAAGCATGTG
CYCLO B R	GCTCTCCACCTTCCGCACCA

ChIP & MEDIP primer sequences

a F	GCTGGACTTAAATGCTGTTGTGCC
a R	AGCTGGAACTCAGGAAGGCTATGA
b F	GAGGAACTGGGCTGTTAGGGATTT
b R	TAACCCTGGCTGAGGAAGAGCAA
c F	AGTGGCTGATTACTGAGCGGTTCT
c R	AAACTAACAGGAGACTGGGACC
d F	ATTATCTGGGCCTGGAGTGTGCAA
d R	TTCCCACACGTCTGCAATCAAAGG
e F	AGACTAGCTGCACCTCCTCATT
e R	TGAGGTCTCGGTGAAACCACCT
f F	AAGAGTAGTTAACAGCCGGAAGCG
f R	AAACTCTGCCCAAGAGGATAAGGG
g F	GCTCTAGGCCGTGGAATCTGG
g R	TCACCGGGCCCACCT
h F	CTGCCTTTACAGCTGCAGCAAGAT
h R	TTTATTTCATGCTCCCAAGGCGGG

PCR primer sequences

MOUSE Set 2 F	AGACCGAGGCCCAGTACAAGTTTA
MOUSE Set 2 R	AACCACCAACATCCAGTTGCGAAG
MOUSE Set 3 F	AGGAAGTGATCTGGGCATTCGTCT
MOUSE Set 3 R	CCATTGTGTTCTTCCAGTGGGCAA
HUMAN Set 2 F	AGGAGAAAGTGAAGAAGCAGCGGT
HUMAN Set 2 R	TGTATAGAAAGGGCGTTGCTGGGA
HUMAN Set 3 F	AGGAGAAAGTGAAGAAGCAGCGGT
HUMAN Set 3 R	AGATCCCTGGCTCCCATC

RACE primer sequences

HUMAN 5' RACE 1	ACCTTGGGTCAGGCAGCTTCAG
HUMAN 5' RACE 2	ACACCGATTTACCCACCCTCAT
HUMAN 3' RACE 1	TCTTGAGCTGAGAGCGTTGCACA
HUMAN 3' RACE 2	CTGCCTTTACAGCTGCAGCAAGAT
MOUSE 3' RACE	AAGACGCTGGCTTCCGTTGTCTTT

CRISPR primer sequences

1 F	GCAAGCAAAGGGTGAGAATCG
1 R	CTCATCCCTGCTTGGGAATGG
2 F	GGTGATCCAGGCGTTCTC
2 R	CACGAATTGCAGCATCTTACC
3 F	GCAGTGCGCATGGACGA
3 R	CACGGGCAGCACGAATTG
4 F	CCCAGGATGGTGAGGTAAG
4 R	TCTCGGTGAAACCACCTG

Supplementary Table 2

Sequences of the primers used for qPCR, PCR, ChIP, MEDIP, RACE and CRISPR-Cas9 experiments described in this study, as indicated.

3C primer sequences

**PROBES**	
3C-probe-1	5'FAM-TCCTGAGAGTTGGCCCTCCCTTGT-3'TAM
3C-probe-2	5'FAM-CTGTGTCCTTGGCTCCACTGCCTT-3'TAM
**PRIMERS (5'->3')**	
3C-primer-1	CACATGGGTGGTTCACATACA
3C-primer-2	TAGGCTGAGATTCTCCCTTGA
3C-primer-3	TCACACCCAAGCTCACATTT
3C-primer-4	TTTCCCTCTCACCCTCTCTC
3C-primer-5	GAGGTACAGATACCACAAGACATT
3C-primer-6	AGGAGGAAGTCCACCCATAC
3C-primer-7	GCTCCAAGGTCCAATGTAGG
3C-primer-8	ATAAGCCACAGAGCCCAGAC
3C-primer-9	GGAACATCAAGGCCTTTCCTC
3C-primer-9b	GGCGCATTTATTATTGTCTGGAAC
3C-primer-10	CAGGGCTTCAAAGAACAGAGA
3C-primer-11	TTATTCCGCTGCCCTTTACTC
3C-primer-12	ACGCACAAGAAACGTCCA
3C-primer-13	CCTTAGAGTGCGACTTTATCCC
3C-primer-14	GGTTGATCTGGTCCAGGAAG
3C-primer-15	CACTTCCTTCCTGTAACGTCTAA
3C-primer-16	GCTGGGAAGCACAGAATGA
3C-primer-16b	CAGTGGAGCCAAGGACAC
3C-primer-17	CAGAGGTTGTTGGTCAGTAGTC
3C-primer-18	TTCACACACCCTCCATTTCTC
3C-primer-19	AGATGCAGGAGGAGGAAAGA
3C-primer-20	TTGAGCCCAGACCTTACCT
3C-primer-21	ATTGTGTATGTGCCGCTACC
3C-primer-22	GGCTGGTCACAGTAAGTAGAAA
3C-primer-23	TGGGAACTCCTAGATAGCATACA
3C-primer-24	GGGTCCACAAGATTATTTGCATAAG
**Primers for restriction efficiency assessment (5'->3')**	
3C-primer-1-RE	ACCATCCTCTCCCTTCTTACA
3C-primer-2-RE	GGACTTCCAGATCTTTACCACATTA
3C-primer-3-RE	AGGCCCACTCTTAGGAATCA
3C-primer-4-RE	CTGAACTGAGTGGTACAGAAAGA
3C-primer-5-RE	GCCCTCTACAGAGATGAAAGAG
3C-primer-6-RE	CAACCACTACCTGGTCATCTG
3C-primer-7-RE	GTAGAACCTGCGATCAAGAGAG
3C-primer-8-RE	ATCCCTGAAGAGCCCAGA
3C-primer-9-RE	GCTGAAGGCGCTGTTTCT
3C-primer-10-RE	GCATTGGCAAGGTCTTAACTATT
3C-primer-11-RE	GGCTCTGAATGCTCCTCAT
3C-primer-12-RE	TGGGTCTTACCTTCCCTGA
3C-primer-13-RE	GGGTCAAGTTCCTTCCTTTCTG
3C-primer-14-RE	GATTCGGGAGATCTGGCATTAC
3C-primer-15-RE	CACACACATTCACACACTTCTTG
3C-primer-16-RE	AAGGAGAAGAGCAAGGGTTC
3C-primer-17-RE	TCTCTCGGCAGTAACCTTCA
3C-primer-18-RE	AAGGAAGCAGCTCCTGTTG
3C-primer-19-RE	GGGAGAGAGTCAGGGAGAC
3C-primer-20-RE	CCCAGCAGTGATCCTATTGTTT
3C-primer-21-RE	GTCAGGAATGCTAGTGGTGAAA
3C-primer-22-RE	AATAGCTCAGTTAGGGCTCTTG
3C-primer-23-RE	TCCCAAAGTGCTGGGATTAC
3C-primer-24-RE	ACTGAACTGGACGCCTCATA

Supplementary Table 3
Sequences of the primers used for the 3C experiments.
